# Bismuth(III)-Catalyzed Regioselective Selenation of Indoles with Diaryl Diselenides: Synthesis of 3-Selanylindoles

**DOI:** 10.3390/molecules29133227

**Published:** 2024-07-08

**Authors:** Mio Matsumura, Airi Umeda, Yuika Sumi, Naoki Aiba, Yuki Murata, Shuji Yasuike

**Affiliations:** School of Pharmaceutical Sciences, Aichi Gakuin University, 1-100 Kusumoto-cho, Chikusa-ku, Nagoya 464-8650, Japan; m-matsu@dpc.agu.ac.jp (M.M.); y-murata@dpc.agu.ac.jp (Y.M.)

**Keywords:** regioselective selenation, bismuth catalyst, bismuth(III) iodide, indole, diaryl diselenide

## Abstract

Heterocyclic aryl selenides have recently attracted considerable research interest owing to their applications in biological and pharmaceutical fields. Herein, we describe a simple and general synthesis of 3-selanylindoles via a novel regioselective C–H selenation of indoles using a bismuth reagent as a catalyst. The reactions of indoles with diselenides in the presence of 10 mol% BiI_3_ at 100 °C in DMF afforded the corresponding 3-selanylindoles in moderate-to-excellent yields. The reaction proceeded efficiently under aerobic conditions by adding only a catalytic amount of BiI_3_, which was non-hygroscopic and less toxic, and both selanyl groups of the diselenide were transferred to the desired products.

## 1. Introduction

**Figure 1:**
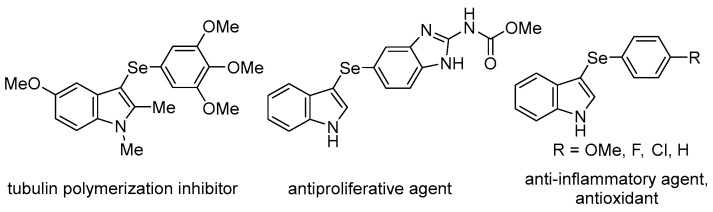
Biologically active 3-selanylindoles.

**Scheme 1:**
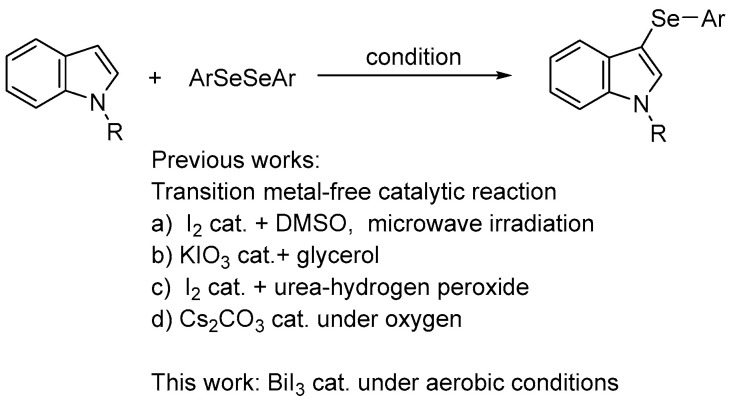
Selenation of indoles with diaryl diselenides.

Organoselenium compounds have received considerable attention in organic chemistry, as well as in biological and pharmaceutical sciences [1,2,3,4,5,6,7,8,9,10,11,12,13,14], and there is growing interest in biologically active unsymmetrical diaryl selenides containing heterocyclic rings (i.e., aryl heteroaryl selenides). For example, 3-selanylindoles, compounds with a selenium side chain substituted at the 3-position of indoles, which are widely used as a basic skeleton in natural products and medicines, have been reported to have biological activities, such as the inhibition of tubulin polymerization, antiproliferative activity, anti-inflammatory properties, and antioxidant activity, and are expected to be used as drug discovery resources (Figure 1) [15,16,17,18,19,20]. Therefore, the development of synthetic methods for these compounds has attracted attention. Direct selenation into indoles has been reported since the 2010s and is a powerful and commonly used method involving the reaction of available indole derivatives with stable and easy-to-handle diselenides as selenium sources. These reactions can be broadly classified into those involving the addition of oxidants [21,22,23] or bases [24,25,26], radical reactions using photoreactors [27,28,29,30,31,32,33,34] or electrolytic devices [35,36], and those using transition metal catalysts containing Pd, Cu, Ag, and Fe [37,38,39,40,41,42]. However, these reactions use excessive reagents, additives, and transition metal catalysts of toxicological concern even in catalytic reactions, and require special equipment and expensive photocatalysts or supporting electrolytes for the photoreactions and electrolytic reactions, respectively. Recently, four transition metal-free catalytic reactions were reported (Scheme 1). Braga et al. developed a catalytic reaction using DMSO as the oxidant in the presence of a catalytic quantity of I_2_; however, the reaction required microwave irradiation [43]. The researchers also used KIO_3_ as a catalyst, but this reaction required an excess (4 equiv.) of glycerol [44]. Roehrs et al. reported an I_2_-catalyzed reaction that required the addition of stoichiometric amounts of urea hydrogen peroxide as an oxidant [45]. Jana et al. developed a reaction using Cs_2_CO_3_ as a catalyst, albeit in an oxygen atmosphere [46]. As mentioned above, catalytic reactions require additives; otherwise, the reaction conditions are restrictive.

Inorganic bismuth compounds have attracted attention in the field of organic synthesis since the 1980s because of their excellent reactivity as mild Lewis acids, nontoxicity, and environmental friendliness [47,48,49,50,51,52]. For example, BiCl_3_, a trivalent bismuth halide, has been reported to act as a catalyst for the following reactions: the Mukaiyama aldol reaction [53,54], the nucleophilic opening of epoxide [55], deoxygenative allylation [56], the Diels–Alder reaction [57,58], the three-component reaction of aldehydes, amines, and ketones or trimethylsilyl cyanide [59,60], the Friedel–Crafts reaction [61], the oxy-Michael addition [62], the aminooxygenation of propargyl amidine [63], and the tandem cyclization of tryptamine-ynamide [64]. More recently, BiCl_3_ has been utilized in the catalytic coupling reactions of aryl iodides or aminobenzimidazoles with arylboronic acids for C(Ar)–C(Ar) and C(Ar)–N bond formation [65,66]. By contrast, bismuth iodide (BiI_3_) is widely used in semiconductors and solar cell devices [67,68]. However, its chemical reactivity in organic reactions is largely unknown, and its use in catalytic reactions has been limited to the deprotection of acetals, guanylation with desulfurization using thioureas and amines, and S,S-acetalization of benzaldehyde [69,70,71]. Inspired by these reports, we present a facile Bi(III)-catalyzed regioselective C(Ar)–Se bond formation reaction of indoles with diaryl diselenides using BiI_3_ as the catalyst for the synthesis of 3-selanylindoles under mild conditions. The system was simple, containing only substrates and a Bi catalyst.

## 2. Results and Discussion

**Figure 2:**
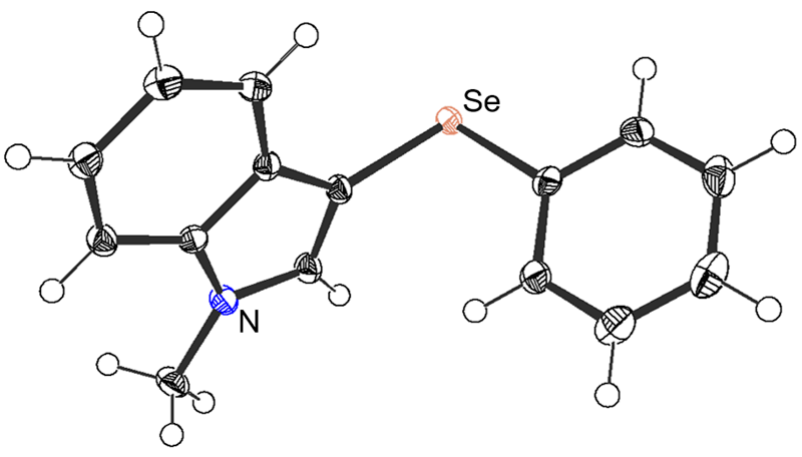
ORTEP drawing of **3aa** with 50% probability (CCDC 2291058).

We initially focused on determining the optimal experimental conditions, including screening for suitable catalysts and solvents, for the synthesis of 3-selanylindole **3aa** using *N*-methylindole **1a** and diphenyl diselenide **2a** as model substrates, the results of which, are summarized in Table 1. *N*-methylindole **1a** (0.5 mmol) was reacted with **2a** (0.25 mmol) in the presence of several Bi catalysts (0.05 mmol) in DMF at 100 °C under aerobic conditions (entries 1–7). BiCl_3_, BiBr_3_, BiI_3_, and Bi(OTf)_3_, which function as Lewis acids, afforded the corresponding 3-selanylindole **3aa** in good-to-excellent yields (77–97%). BiI_3_ displayed the best yield and reaction time, and both selanyl groups were efficiently transferred from the diselenide to product **3aa** (entry 3). Furthermore, although bismuth halides such as BiCl_3_ and BiBr_3_ are hygroscopic, BiI_3_ can be easily handled in air without such concerns. By contrast, antimony catalysts with the same group of atoms as bismuth and other Lewis acid catalysts were less effective than BiI_3_ (entries 8–12). A comparison to iodine (I_2_) was also attempted; however, the reaction barely progressed (entry 13). Solvent screening indicated that the reaction proceeded efficiently in DMF (97%), DMSO (89%), and THF (60%), whereas CH_3_CN, MeOH, dioxane, 1,2-DCE, and toluene were inefficient (entries 3 and 14–20). When the reaction was performed at 60 °C, the reaction time increased markedly to 8 h (entry 21). The reaction performed under oxygen produced **3aa** in a high yield (94%), which was almost identical to that obtained under aerobic conditions (entries 3 and 22). However, the yield was notably suppressed (9%) under an argon atmosphere (entry 23). Decreasing the BiI_3_ loading from 10 to 5 and 1 mol% markedly prolonged the reaction time, although the reaction afforded the desired product (entries 24 and 25). The best result was obtained under aerobic conditions at 100 °C when **1a** was treated with 0.5 equivalents of diselenide **2a** in the presence of BiI_3_ (10 mol%) in DMF (entry 3). This selenation could also be scaled up to 10 mmol. The desired product **3aa** was obtained in an excellent yield (99%), generating up to 2.84 g of the product. Furthermore, the reaction of **1a** and **2a** with 1 equivalent of TEMPO [(2,2,6,6-tetramethylpiperidin-1-yl)oxyl] or 1,1-diphenylethylene as radical scavengers afforded **3aa** in yields of 94% and 96%, respectively (entries 26 and 27). These results indicate that the reaction system does not follow a radical mechanism. The regiochemistry of 3-selanylindole **3aa** was elucidated using ^1^H-NMR and single-crystal X-ray analyses (Figure 2). The ^1^H-NMR spectrum of **3aa** was consistent with that of the standard sample [41].

**Figure 3:**
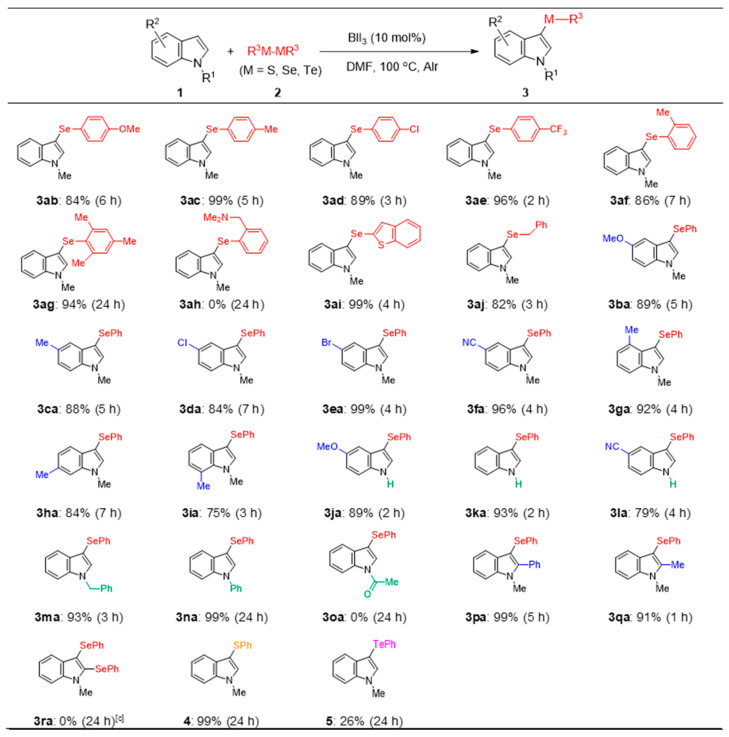
Substrate scope: reaction of indoles with dichalcogenides ^[a,b]^. ^[a]^ **1** (0.5 mmol), **2** (0.25 mmol), BiI_3_ (0.05 mmol), and DMF (2 mL). ^[b]^ Yield of isolated products. ^[c]^ **2a** (0.5 mmol); **3aa** was isolated in a yield of 98%.

To understand the scope and limitations of the developed regioselective selenation reaction, various indoles **1** (0.5 mmol) were reacted with diselenides **2** (0.25 mmol) under the optimized conditions (Figure 3). The reaction of *N*-methylindole **1a** with diaryl diselenides **2b**–**i** afforded the corresponding products, i.e., **3ab**–**ai**, in good-to-excellent yields, except for **3ah**. For **3ab**–**ae**, the presence of an electron-donating or electron-withdrawing group at the 4-position of the benzene ring of diselenides **3b**–**e** did not affect the reaction progression, although the reaction time was slightly prolonged when electron-donating groups were substituted. Sterically hindered *ortho*-substituted diselenides **2f** and **2g** reacted to give selenides **3af** and **3ag**, respectively. By contrast, for **2h**, which comprises a benzylamino group, the reaction did not proceed, and the starting materials were recovered. For the reaction using diaryl diselenide **2i**, which bears a heterocyclic ring, **3ai** was afforded in a good yield. Dibenzyl diselenide **2j**, which contains a benzyl moiety as the alkyl group, also afforded **3aj** in a good yield (82%). Next, the reaction of diphenyl diselenide **2a** with various *N*-methylindoles, i.e., **1b**–**i**, bearing electron-donating or electron-withdrawing groups on the benzene ring afforded the desired products **3ba**–**3ia** in satisfactory yields (75–99%). The reaction proceeded smoothly from the unsubstituted indoles **1j**–**l** to obtain the parent 3-selanylindoles **3ja**–**la** (79–93%). Furthermore, the reaction of the *N*-substituted indoles **1m** and **1n** with benzyl or phenyl groups on the nitrogen also gave the corresponding products **3ma** and **3na**; however, *N*-acetylindole **1o** with an electron-withdrawing group did not give **3oa**, and the starting materials were recovered. These results suggest that the reaction is electrically influenced by the substituents on the indole nitrogen. 2-Phenyl- and 2-methylindoles **1p** and **1q** were treated with **2a** to afford the 3-selanyl-2-substituted indoles **3pa** and **3qa**, respectively. The attempted double selenation of **1a** using two equivalents of diphenyl diselenide **2a** did not yield the corresponding 2,3-diselanylindole **3ra**; instead, 3-selanylindole **3aa** was isolated in a yield of 98%. These results suggest that this reaction proceeds only at the 3-position of the indole. Finally, the reaction of **1a** with dichalcogenides containing sulfur and tellurium was attempted. The reaction with diphenyl disulfide afforded the desired 3-sulfanylindole **4** in an excellent yield (99%), although the reaction time (24 h) was longer than that with diselenide, which is a selenium reagent. By contrast, the reaction proceeded to a certain extent with diphenyl ditelluride, and indole **5** was obtained in a yield of 26%.

**Scheme 2:**
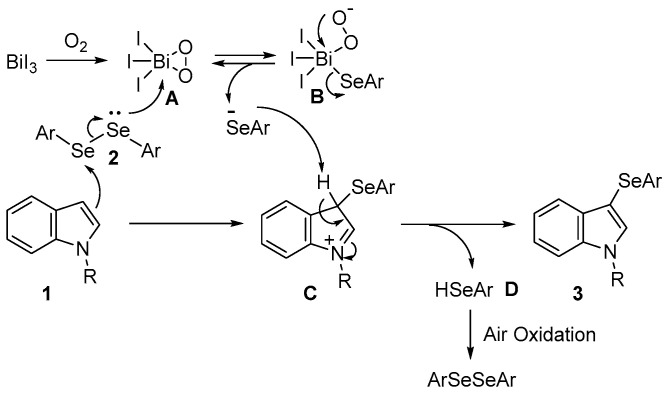
Possible mechanism.

However, the reaction mechanism for this selenation remains unclear. Circumstantial evidence indicates that the reaction was affected by the gaseous atmosphere and proceeded smoothly in the presence of a molecular oxygen atmosphere while being notably suppressed in an inert gas atmosphere (Table 1: entries 3, 22, and 23). BiI_3_ forms a pentacoordinated complex with bismuth, the central atom, and the oxygen atoms of reagents and solvents such as Mo_8_O_26_ and THF [72,73]. Therefore, a possible mechanism for this reaction is illustrated in Scheme 2. The initial step was the generation of the pentacoordinated Bi–peroxo complex **A** from BiI_3_ and oxygen. While the selenium atom of the diselenide coordinates with complex **A**, the 3-position of the indole nucleophilically attacks another selenium atom, forming complex **B** and intermediate **C**. The aryl selenide anion formed during the interconversion between complexes **B** and **A** attacks intermediate **C** to form 3-selanylindole **3** and selenol **D**. Selenol **D** is converted to diselenide **2** via oxidation in air. Therefore, the reaction proceeds with 0.5 equivalents of diselenide, and both selanyl groups are used for the reaction. Bismuth complexes **A** and **B**, which are expected to form during this process, have not yet been confirmed or isolated.

## 3. Conclusions

Herein, we report a simple Bi-catalyzed regioselective selenation protocol for the synthesis of 3-selanylindoles under mild reaction conditions. The reaction is atom-economical, with the participation of both selanyl groups of the diaryl diselenide. Indoles and diselenides bearing different functional groups afforded the corresponding products in satisfactory yields. This reaction is the first example of the Bi-catalyzed C–H selenation of aromatic heterocycles. Detailed studies on the exact mechanism of this reaction and the synthesis of asymmetric selenides containing other heterocyclic rings using this protocol are currently underway.

## 4. Materials and Methods

### 4.1. General Information

All the chemicals, including organic solvents, were obtained from commercial vendors and used as received without further purification. All chromatographic separations were accomplished with Silica Gel 60N (Kanto Chemical Co., Inc., Tokyo, Japan). Thin-layer chromatography (TLC) was performed using Macherey–Nagel Pre-coated TLC plates Sil G25 UV_254_. Melting point measurements were conducted on a Yanagimoto micro-melting point hot-stage apparatus (MP-S3) and reported as uncorrected values. In addition, ^1^H NMR (TMS: *δ* = 0.00 ppm as an internal standard), ^13^C NMR (CDCl_3_: *δ* = 77.00 ppm as an internal standard), ^19^F NMR (376 MHz, benzotrifluoride; *δ* = −64.0 ppm as an external standard), and ^77^Se NMR (76 MHz, diphenyldiselenide; *δ* = 463.15 ppm as an external standard) spectra were recorded on JEOL ECZ-400S (400, 100, 376, and 76 MHz for ^1^H-, ^13^C-, ^19^F-, and ^77^Se NMR, respectively) spectrometers (JEOL Ltd., Tokyo, Japan). GC-MS (EI) spectra were recorded on Agilent 5977 E Diff-SST MSD-230 V spectrometer. HRMS (ESI) spectra were recorded on Agilent 6230 (Agilent Technologies Japan, Ltd., Tokyo, Japan). X-ray measurements were recorded on Rigaku XtaLAB Synergy with a HyPix3000 diffractometer (Rigaku, Corp., Tokyo, Japan). IR spectra were recorded on an FTIR-8400S or IRAffinity-1S system from a Shimadzu spectrometer (SHIMADZU Corp, Kyoto, Japan) and are reported as the frequencies of absorption (cm^−1^). Only selected IR absorbencies are reported. The spectroscopic data of the calcogenated indoles **3aa**–**ad**, **3ag**, **3ka**, **3ma**, **3qa**, **4** [41], **3aj** [74], **3ba** [42], **3ea**, **3ja**, **3na** [35], **3la** [27], **3pa** [75], and **5** [76] are in accordance with those in the literature, and their characterization data are in Supplementary Materials.

### 4.2. General Procedure for the Synthesis of Calcogenated Indoles

The indole derivative (**1**) (0.5 mmol) was added to a solution of dichalcogenide (**2**) (0.25 mmol, 0.5 eq.) and bismuth(III) iodide (30 mg, 0.05 mmol, and 10 mol%) in anhydrous dimethylformamide (2 mL). After stirring at 100 °C in an oil bath, the mixture was cooled to room temperature and evaporated to dryness under reduced pressure. The crude product was purified on a silica gel column chromatography to give the desired product **3**.

### 4.3. Characterization Data of Novel Compounds

#### 4.3.1. 3-(4-Trifluoromethylphenyl)selanyl-1-methyl-1H-indole (**3ae**)

Yield: 171 mg (96%); Colorless prism (from CH_2_Cl_2_-Hexane); m.p. 133.0–135.0 °C; *R_f_* = 0.54 (CH_2_Cl_2_-Hexane, 1:2). ^1^H NMR (400 MHz, CDCl_3_): *δ* = 7.57 (d, *J* = 7.8 Hz, 1H; Ar-H), 7.41 (d, *J* = 8.2 Hz, 1H; Ar-H), 7.36 (s, 1H; Ar-H), 7.35–7.30 (m, 3H; Ar-H), 7.26 (d, *J* = 8.2 Hz, 2H; Ar-H), 7.19 (td, *J* = 8.2, 0.9 Hz, 1H; Ar-H), 3.88 ppm (s, 3H; *N*-CH_3_). ^13^C NMR (100 MHz, CDCl_3_): *δ* = 140.0 (C), 137.5 (C), 135.9 (CH), 130.4 (C), 128.0 (CH), 127.5 (q, *J* = 32 Hz, C), 125.5 (q, *J* = 3.9 Hz, CH), 124.2 (q, *J* = 272 Hz, C), 122.7 (CH), 120.7 (CH), 120.2 (CH), 109.7 (CH), 94.6 (C), 33.2 ppm (CH_3_). ^19^F NMR (376 MHz, CDCl_3_): *δ* = −63.7 ppm. ^77^Se NMR (76 MHz, CDCl_3_): *δ* = 223.4 ppm. IR (ATR): ν^~^ = 739, 822, 1072, 1105, 1321 cm^−1^. MS (EI, 70 eV): *m*/*z* (%) = 355 (21) [M]^+^, 275 (100), 130 (14). HRMS (ESI): *m*/*z* calcd for C_16_H_12_F_3_NSe: 355.0087 [M]^+^; found: 355.0088.

#### 4.3.2. 1-Methyl-3-(2-methylphenyl)selanyl-1H-indole (**3af**)

Yield: 129 mg (86%); Colorless plate (from CH_2_Cl_2_-Hexane); m.p. 129.0–132.0 °C; *R_f_* = 0.20 (CH_2_Cl_2_-Hexane, 1:5). ^1^H NMR (400 MHz, CDCl_3_): *δ* = 7.59 (d, *J* = 7.8 Hz, 1H; Ar-H), 7.39 (d, *J* = 8.2 Hz, 1H; Ar-H), 7.31 (s, 1H; Ar-H), 7.30 (td, *J* = 8.2, 0.9 Hz, 1H; Ar-H), 7.17 (td, *J* = 7.3, 0.9 Hz, 1H; Ar-H), 7.10 (d, *J* = 7.3 Hz, 1H; Ar-H), 7.00 (td, *J* = 7.8, 2.3 Hz, 1H; Ar-H), 6.86–6.80 (m, 2H; Ar-H), 3.85 (s, 3H; *N*-CH_3_), 2.46 ppm (s, 3H; CH_3_). ^13^C NMR (100 MHz, CDCl_3_): *δ* = 137.6 (C), 135.92 (C), 135.87 (CH), 134.8 (C), 130.8 (C), 129.7 (CH), 127.8 (CH), 126.4 (CH), 125.2 (CH), 122.4 (CH), 120.5 (CH), 120.4 (CH), 109.5 (CH), 94.9 (C), 33.1 (CH_3_), 21.2 ppm (CH_3_). ^77^Se NMR (76 MHz, CDCl_3_): *δ* = 178.3 ppm. IR (ATR): ν^~^ = 411, 426, 729, 746, 1456 cm^−1^. MS (EI, 70 eV): *m*/*z* (%) = 301 (40) [M]^+^, 221 (60), 131 (100), 91 (30). HRMS (ESI): *m*/*z* calcd for C_16_H_15_NSe: 301.0370 [M]^+^; found: 301.0370.

#### 4.3.3. 3-(2-Benzothienyl)selanyl-1-methyl-1H-indole (**3ai**)

Yield: 171 mg (99%); Yellow needle (from CH_2_Cl_2_-Hexane); m.p. 144.0–147.0 °C; *R_f_* = 0.21 (CH_2_Cl_2_-Hexane, 1:5). ^1^H NMR (400 MHz, CDCl_3_): *δ* = 7.78 (d, *J* = 7.8 Hz, 1H; Ar-H), 7.62 (d, *J* = 6.9 Hz, 1H; Ar-H), 7.60 (d, *J* = 7.3 Hz, 1H; Ar-H), 7.39 (s, 1H; Ar-H), 7.36 (d, *J* = 7.8 Hz, 1H; Ar-H), 7.32–7.16 (m, 5H; Ar-H), 3.83 ppm (s, 3H; *N*-CH_3_). ^13^C NMR (100 MHz, CDCl_3_): *δ* = 142.2 (C), 140.5 (C), 137.2 (C), 135.1 (CH), 132.1 (C), 130.3 (C), 126.3 (CH), 124.1 (CH), 123.5 (CH), 122.51 (CH), 122.47 (CH), 121.5 (CH), 120.5 (CH), 120.3 (CH), 109.6 (CH), 97.1 (C), 33.1 ppm (CH_3_). ^77^Se NMR (76 MHz, CDCl_3_): *δ* = 179.9 ppm. IR (ATR): ν^~^ = 426, 486, 556, 723, 735, 1236 cm^−1^. MS (EI, 70 eV): *m*/*z* (%) = 343 (14) [M]^+^, 263 (100), 207 (21), 131 (43), 89 (21), 44 (29). HRMS (ESI): *m*/*z* calcd for C_17_H_13_NSSe: 342.9934 [M]^+^; found: 342.9932.

#### 4.3.4. 1,5-Dimethyl-3-phenylselanyl-1H-indole (**3ca**)

Yield: 133 mg (88%); Colorless plate (from CH_2_Cl_2_-Hexane); m.p. 104.0–105.0 °C; *R_f_* = 0.58 (CH_2_Cl_2_-Hexane, 1:5). ^1^H NMR (400 MHz, CDCl_3_): *δ* = 7.41 (s, 1H; Ar-H), 7.27–7.20 (m, 4H; Ar-H), 7.14–7.05 (m, 4H; Ar-H), 3.81 (s, 3H; *N*-CH_3_), 2.43 ppm (s, 3H; CH_3_). ^13^C NMR (100 MHz, CDCl_3_): *δ* = 135.83 (C), 135.76 (CH), 134.5 (C), 130.9 (C), 129.8 (C), 128.9 (CH), 128.3 (CH), 125.4 (CH), 124.1 (CH), 120.0 (CH), 109.2 (CH), 95.0 (C), 33.1 (CH_3_), 21.4 ppm (CH_3_). ^77^Se NMR (76 MHz, CDCl_3_): *δ* = 206.4 ppm. IR (ATR): ν^~^ = 424, 457, 731, 793, 1474, 1506 cm^−1^. MS (EI, 70 eV): *m*/*z* (%) = 301 (20) [M]^+^, 221 (100), 144 (13). HRMS (ESI): *m*/*z* calcd for C_16_H_15_NSe: 301.0370 [M]^+^; found: 301.0369.

#### 4.3.5. 5-Chloro-1-methyl-3-phenylselanyl-1H-indole (**3da**)

Yield: 135 mg (84%); Colorless plate (from CH_2_Cl_2_-Hexane); m.p. 132.0–133.5 °C; *R_f_* = 0.54 (CH_2_Cl_2_-Hexane, 1:5). ^1^H NMR (400 MHz, CDCl_3_): *δ* = 7.60 (s, 1H; Ar-H), 7.33 (s, 1H; Ar-H), 7.28–7.19 (m, 4H; Ar-H), 7.15–7.08 (m, 3H; Ar-H), 3.82 ppm (s, 3H; *N*-CH_3_). ^13^C NMR (100 MHz, CDCl_3_): *δ* = 136.9 (CH), 135.9 (C), 133.7 (C), 131.9 (C), 129.0 (CH), 128.6 (CH), 126.5 (C), 125.7 (CH), 122.8 (CH), 119.9 (CH), 110.7 (CH), 95.7 (C), 33.3 ppm (CH_3_). ^77^Se NMR (76 MHz, CDCl_3_): *δ* = 209.6 ppm. IR (ATR): ν^~^ = 422, 457, 689, 733, 795, 1422, 1474 cm^−1^. MS (EI, 70 eV): *m*/*z* (%) = 321 (25) [M]^+^, 241 (100), 164 (15). HRMS (ESI): *m*/*z* calcd for C_15_H_12_ClNSe: 320.9823 [M]^+^; found: 320.9825.

#### 4.3.6. 1-Methyl-3-phenylselanyl-1H-indole-5-carbonitrile (**3fa**)

Yield: 149 mg (96%); Colorless prism (from CH_2_Cl_2_-Hexane); m.p. 198.0–199.5 °C; *R_f_* = 0.34 (CH_2_Cl_2_-Hexane, 1:1). ^1^H NMR (400 MHz, CDCl_3_): *δ* = 7.95 (d, *J* = 1.4 Hz, 1H; Ar-H), 7.49 (dd, *J* = 8.7, 1.4 Hz, 1H; Ar-H), 7.45 (s, 1H; Ar-H), 7.41 (d, *J* = 8.7 Hz, 1H; Ar-H), 7.23–7.20 (m, 2H; Ar-H), 7.17–7.12 (m, 3H; Ar-H), 3.88 ppm (s, 3H; *N*-CH_3_). ^13^C NMR (100 MHz, CDCl_3_): *δ* = 139.0 (C), 137.6 (CH), 132.8 (C), 130.5 (C), 129.11 (CH), 129.07 (CH), 126.14 (CH), 126.08 (CH), 125.4 (CH), 120.4 (C), 110.5 (CH), 103.6 (C), 97.9 (C), 33.3 ppm (CH_3_). ^77^Se NMR (76 MHz, CDCl_3_): *δ* = 212.5 ppm. IR (ATR): ν^~^ = 461, 474, 631, 692, 741 cm^−1^. MS (EI, 70 eV): *m*/*z* (%) = 312 (17) [M]^+^, 232 (100), 155 (16). HRMS (ESI): *m*/*z* calcd for C_16_H_12_N_2_Se: 312.0166 [M]^+^; found: 312.0166.

#### 4.3.7. 1,4-Dimethyl-3-phenylselanyl-1H-indole (**3ga**)

Yield: 138 mg (92%); Colorless plate (from CH_2_Cl_2_-Hexane); m.p. 84.0–85.0 °C; *R_f_* = 0.25 (CH_2_Cl_2_-Hexane, 1:5). ^1^H NMR (400 MHz, CDCl_3_): *δ* = 7.29 (s, 1H; Ar-H), 7.24–7.18 (m, 4H; Ar-H), 7.15–7.11 (m, 2H; Ar-H), 7.07 (tt, *J* = 6.9, 1.4 Hz, 1H; Ar-H), 6.89 (d, *J* = 7.3 Hz, 1H; Ar-H), 3.80 (s, 3H; *N*-CH_3_), 2.69 ppm (s, 3H; CH_3_). ^13^C NMR (100 MHz, CDCl_3_): *δ* = 137.9 (C), 136.8 (CH), 136.4 (C), 132.3 (C), 129.0 (CH), 127.9 (CH), 125.2 (CH), 122.4 (CH), 122.0 (CH), 107.5 (CH), 94.6 (C), 33.1 (CH_3_), 18.7 ppm (CH_3_). ^77^Se NMR (76 MHz, CDCl_3_): *δ* = 251.5 ppm. IR (ATR): ν^~^ = 457, 667, 689, 727, 739, 1474 cm^−1^. MS (EI, 70 eV): *m*/*z* (%) = 301 (33) [M]^+^, 221 (100), 144 (44). HRMS (ESI): *m*/*z* calcd for C_16_H_15_NSe: 301.0370 [M]^+^; found: 301.0369.

#### 4.3.8. 1,6-Dimethyl-3-phenylselanyl-1H-indole (**3ha**)

Yield: 126 mg (84%); Colorless plate (from CH_2_Cl_2_-Hexane); m.p. 86.0–87.5 °C; *R_f_* = 0.45 (CH_2_Cl_2_-Hexane, 1:5). ^1^H NMR (400 MHz, CDCl_3_): *δ* = 7.50 (d, *J* = 8.2 Hz, 1H; Ar-H), 7.26–7.22 (m, 3H; Ar-H), 7.18 (s, 1H; Ar-H), 7.14–7.06 (m, 3H; Ar-H), 7.01 (d, *J* = 7.8 Hz, 1H; Ar-H), 3.80 (s, 3H; *N*-CH_3_), 2.52 ppm (s, 3H; CH_3_). ^13^C NMR (100 MHz, CDCl_3_): *δ* = 137.8 (C), 135.1 (CH), 134.3 (C), 132.4 (C), 128.9 (CH), 128.5 (C), 128.4 (CH), 125.4 (CH), 122.1 (CH), 120.1 (CH), 109.5 (CH), 95.6 (C), 32.9 (CH_3_), 21.8 ppm (CH_3_). ^77^Se NMR (76 MHz, CDCl_3_): *δ* = 209.3 ppm. IR (ATR): ν^~^ = 430, 598, 689, 729, 797 cm^−1^. MS (EI, 70 eV): *m*/*z* (%) = 301 (19) [M]^+^, 221 (100), 144 (17). HRMS (ESI): *m*/*z* calcd for C_16_H_15_NSe: 301.0370 [M]^+^; found: 301.0371.

#### 4.3.9. 1,7-Dimethyl-3-phenylselanyl-1H-indole (**3ia**)

Yield: 113 mg (75%); Colorless plate (from CH_2_Cl_2_-Hexane); m.p. 110.0–111.0 °C; *R_f_* = 0.34 (CH_2_Cl_2_-Hexane, 1:5). ^1^H NMR (400 MHz, CDCl_3_): *δ* = 7.46 (d, *J* = 7.8 Hz, 1H; Ar-H), 7.24–7.20 (m, 3H; Ar-H), 7.13–7.05 (m, 3H; Ar-H), 7.01 (t, *J* = 7.3 Hz, 1H; Ar-H), 6.96 (d, *J* = 6.9 Hz, 1H; Ar-H), 4.08 (s, 3H; *N*-CH_3_), 2.79 ppm (s, 3H; CH_3_). ^13^C NMR (100 MHz, CDCl_3_): *δ* = 137.2 (CH), 136.1 (C), 134.2 (C), 131.8 (C), 128.9 (CH), 128.5 (CH), 125.4 (CH), 125.1 (CH), 121.5 (C), 120.6 (CH), 118.7 (CH), 95.7 (C), 37.0 (CH_3_), 19.6 ppm (CH_3_). ^77^Se NMR (76 MHz, CDCl_3_): *δ* = 208.8 ppm. IR (ATR): ν^~^ = 689, 733, 748, 781, 1450 cm^−1^. MS (EI, 70 eV): *m*/*z* (%) = 301 (18) [M]^+^, 221 (100), 144 (17). HRMS (ESI): *m*/*z* calcd for C_16_H_15_NSe: 301.0370 [M]^+^; found: 301.0370.

### 4.4. Single-Crystal X-ray Diffraction Experiment of ***3aa***

A suitable crystal was selected and measured on an XtaLAB Synergy, Single source at home/near, HyPix3000 diffractometer. The crystal was kept at 103 K in an N_2_ cold stream during data collection. Using Olex2 [77], the structure was solved with the SHELXT [78] structure solution program using Intrinsic Phasing and refined with the SHELXL [79] refinement package using Least Squares minimization. Crystal Data for **3aa**: C_15_H_13_NSe (*M* = 286.22 g/mol), monoclinic, space group *P*2_1_/n (no. 14), *a* = 7.73810(10) Å, *b* = 9.03610(10) Å, *c* = 18.1620(2) Å, *β* = 101.9160(10)°, *V* = 1242.56(3) Å^3^, *Z* = 4, *T* = 103 K, *μ*(Cu Kα) = 3.873 mm^−1^, *D_calc_* = 1.530 g/cm^3^, 6253 reflections measured (9.954° ≤ 2*Θ* ≤ 136.378°), and 2263 unique reflections (*R*_int_ = 0.0255 and *R*_sigma_ = 0.0218), which were used in all calculations. The final *R*_1_ was 0.0243 (I > 2σ(I)), and *wR*_2_ was 0.0652 (all data).

## Figures and Tables

**Table 1:** Optimization of the reaction conditions ^[a]^.

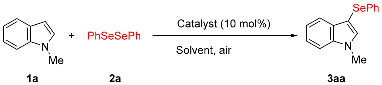					
Entry	Catalyst	Solvent	Temp. [°C]	Time [h]	Yield (%) ^[b]^
1	BiCl_3_	DMF	100	24	85
2	BiBr_3_	DMF	100	6	84
3	BiI_3_	DMF	100	1	97 (91) ^[c]^
4	BiF_3_	DMF	100	24	14
5	Bi(OTf)_3_	DMF	100	24	77
6	Bi(ONO_2_)_3_	DMF	100	24	49
7	Ph_3_Bi	DMF	100	24	2
8	SbBr_3_	DMF	100	24	77
9	SbI_3_	DMF	100	24	74
10	AlCl_3_	DMF	100	24	11
11	InCl_3_	DMF	100	24	25
12	FeCl_3_	DMF	100	24	41
13	I_2_	DMF	100	24	20
14	BiI_3_	DMSO	100	2	89
15	BiI_3_	CH_3_CN	80	24	45
16	BiI_3_	THF	60	24	60
17	BiI_3_	MeOH	60	24	51
18	BiI_3_	1,2-DCE	80	2	12
19	BiI_3_	Dioxane	100	24	19
20	BiI_3_	Toluene	100	24	12
21	BiI_3_	DMF	60	8	89
22 ^[d]^	BiI_3_	DMF	100	1	94
23 ^[e]^	BiI_3_	DMF	100	24	9
24 ^[f]^	BiI_3_	DMF	100	8	94
25 ^[g]^	BiI_3_	DMF	100	24	92
26 ^[h]^	BiI_3_	DMF	100	1	94
27 ^[i]^	BiI_3_	DMF	100	1	96

^[a]^ Conditions: **1a** (0.5 mmol), **2a** (0.25 mmol), catalyst (10 mol%), and solvent (2 mL). ^[b]^ GC yield using biphenyl as the internal standard. ^[c]^ Isolated yield. ^[d]^ Under O_2_. ^[e]^ Under argon. ^[f]^ BiI_3_ (5 mol%). ^[g]^ BiI_3_ (1 mol%). ^[h]^ TEMPO (0.5 mmol). ^[i]^ Diphenylethylene (0.5 mmol).

## Data Availability

The data that support the findings of this study are available in the manuscript and Supplementary Materials of this article.

## Supplementary Materials

The following supporting information can be downloaded at: https://www.mdpi.com/article/10.3390/molecules29133227/s1. The characterization data of known compounds and ^1^H- and ^13^C-NMR spectra are available online. The crystal structures have been deposited to the CCDC with the number 2291058, and the CIF files are also provided.
