# Supplementary material for: Bismuth(III)-Catalyzed Regioselective Selenation of Indoles with Diaryl Diselenides: Synthesis of 3-Selanylindoles
Source: Molecules. 2024 Jul 8;29(13):3227. doi: 10.3390/molecules29133227 (PMC11243167; doi:10.3390/molecules29133227)

Supplementary Materials

Bismuth(III)-catalyzed regioselective selenation of indoles with diaryl diselenides: Synthesis of 3-selanylindoles

Mio Matsumura, Airi Umeda, Yuika Sumi, Naoki Aiba, Yuki Murata, and Shuji Yasuike*

[a] *School of Pharmaceutical Sciences, Aichi Gakuin University, 1-100 Kusumoto-cho, Chikusa-ku, Nagoya 464-8650, Japan*

* E-mail: s-yasuik@dpc.agu.ac.jp

Contents

1. Characterization data of known compounds S1

2. References S7

3. Copies of ^{1}H and ^{13}C NMR spectra of products S8

1. Characterization data of known compounds

1-Methyl-3-phenylselanyl-1*H*-indole (**3aa**) [1]

Yield: 130 mg (91%); Colorless prisms (from CH_2Cl_2-Hexane); m.p. 69.0–70.0 °C; R_f = 0.20 (CH_2Cl_2-Hexane, 1:5). ^{1}H NMR (400 MHz, $CDCl_3$): δ = 7.63 (d, J = 7.8 Hz, 1H; Ar-H), 7.38 (d, J = 8.2 Hz, 1H; Ar-H), 7.33 (s, 1H; Ar-H), 7.29 (t, J = 7.3 Hz, 1H; Ar-H), 7.25–7.06 (m, 6H; Ar-H), 3.85 ppm (s, 3H; *N*-CH_3). ^{13}C NMR (100 MHz, $CDCl_3$): δ = 137.4 (C), 135.6 (CH), 134.2 (C), 130.7 (C), 128.9 (CH), 128.5 (CH), 125.5 (CH), 122.4 (CH), 120.44 (CH), 120.39 (CH), 109.5 (CH), 95.9 (C), 33.1 ppm (CH_3). ^{77}Se NMR (76 MHz, $CDCl_3$): δ = 208.3 ppm. MS (EI, 70 eV): *m/z* (%) = 287 (20) $[M]^+$, 207 (100), 165 (10), 130 (10), 77 (5).

3-(4-Methoxylphenyl)selanyl-1-methyl-1*H*-indole (**3ab**) [1]

Yield: 134 mg (84%); Orange oil (from CH_2Cl_2-Hexane); R_f = 0.59 (Hexane-Ether, 10:1). ^{1}H NMR (400 MHz, $CDCl_3$): δ = 7.64 (d, J = 7.8 Hz, 1H; Ar-H), 7.35 (d, J = 8.2 Hz, 1H; Ar-H), 7.30 (s, 1H; Ar-H), 7.29–7.23 (m, 3H; Ar-H), 7.16 (td, J = 8.2, 0.9 Hz, 1H; Ar-H), 6.70 (dt, J = 8.7, 1.8 Hz, 2H; Ar-H), 3.82 (s, 3H; *N*-CH_3), 3.71 ppm (s, 3H; *O*-CH_3). ^{13}C NMR (100 MHz, $CDCl_3$): δ = 137.4 (C), 135.1 (CH), 131.1 (CH), 130.6 (C), 123.8 (C), 122.3 (CH), 120.4 (CH), 120.3 (CH), 114.7 (CH), 109.4 (CH), 97.3 (C), 77.2 (C), 55.2 (CH_3), 33.0 ppm (CH_3). ^{77}Se NMR (76 MHz, $CDCl_3$): δ = 199.8 ppm. MS (EI, 70 eV): *m/z* (%) = 317 (22) $[M]^+$, 237 (100), 222 (90) 130 (36).

1-Methyl-3-(4-methylphenyl)selanyl-1*H*-indole (**3ac**) [1]

Yield: 151 mg (99%); Colorless prism (from CH_2Cl_2-Hexane); m.p. 123.0–125.0 °C; R_f = 0.50 (CH_2Cl_2-Hexane, 1:5). ^{1}H NMR (400 MHz, $CDCl_3$): δ = 7.63 (d, J = 6.9 Hz, 1H; Ar-H), 7.37 (d, J = 8.2 Hz, 1H; Ar-H), 7.32 (s, 1H; Ar-H), 7.28 (t, J = 6.9 Hz, 1H; Ar-H), 7.18–7.13 (m, 3H; Ar-H), 6.94 (d, J = 7.8 Hz, 2H; Ar-H), 3.84 (s, 3H; *N*-CH_3), 2.23 ppm (s, 3H; CH_3). ^{13}C NMR (100 MHz, $CDCl_3$): δ = 137.4 (C), 135.4 (CH), 135.3 (C), 130.7 (C), 130.2 (C), 129.7 (CH), 128.9 (CH), 122.3 (CH), 120.5 (CH), 120.3 (CH), 109.4 (CH), 96.4 (C), 33.0 (CH_3), 20.9 ppm (CH_3). ^{77}Se NMR (76 MHz, $CDCl_3$): δ = 202.9 ppm. MS (EI, 70 eV): *m/z* (%) = 301 (15) $[M]^+$, 221 (100), 130 (10).

3-(4-Chlorophenyl)selanyl-1-methyl-1*H*-indole (**3ad**) [1]

Yield: 142 mg (89%); Colorless prism (from CH_2Cl_2-Hexane); m.p. 125.0–126.5 °C; R_f = 0.50 (CH_2Cl_2-Hexane, 1:5). ^{1}H NMR (400 MHz, $CDCl_3$): δ = 7.58 (dd, J = 6.9, 0.9 Hz, 1H; Ar-H), 7.39 (d, J = 8.2 Hz, 1H; Ar-H) 7.34 (s, 1H; Ar-H), 7.30 (ddd, J = 8.2, 7.3, 1.4 Hz, 1H; Ar-H), 7.18 (ddd, J = 8.2, 7.3, 1.4 Hz, 1H; Ar-H), 7.15–7.12 (m, 2H; Ar-H), 7.08 (dt, J = 9.1, 1.8 Hz, 2H; Ar-H), 3.86 ppm (s, 3H; *N*-CH_3). ^{13}C NMR (100 MHz, $CDCl_3$): δ = 137.5 (C), 135.6 (CH), 132.5 (C), 131.4 (C), 130.4 (C), 129.8 (CH), 128.9 (CH), 122.6 (CH), 120.6 (CH), 120.3 (CH), 109.6 (CH), 95.6 (C), 33.1 ppm (CH_3). ^{77}Se NMR (76 MHz, $CDCl_3$): δ = 212.0 ppm. MS (EI, 70 eV): *m/z* (%) = 321 (15) $[M]^+$, 241 (100), 130 (15).

1-Methyl-3-(2,4,6-trimethylphenyl)selanyl-1*H*-indole (**3ag**) [1]

Yield: 154 mg (94%); Colorless plate (from CH_2Cl_2-Hexane); m.p. 110.0–111.0 °C; R_f = 0.71 (CH_2Cl_2-Hexane, 1:5). ^{1}H NMR (400 MHz, $CDCl_3$): δ = 7.56 (d, J = 7.8 Hz, 1H; Ar-H), 7.27 (d, J = 8.2 Hz, 1H; Ar-H), 7.23 (td, J = 6.9, 0.9 Hz, 1H; Ar-H), 7.11 (td, J = 7.8, 0.9 Hz, 1H; Ar-H), 7.04 (s, 1H; Ar-H), 6.87 (s, 2H; Ar-H), 3.74 (s, 3H; *N*-CH_3), 2.58 (s, 6H; CH_3), 2.23 ppm (s, 3H; CH_3). ^{13}C NMR (100 MHz, $CDCl_3$): δ = 142.4 (C), 137.7 (C), 137.0 (C), 132.7 (CH), 130.2 (C), 129.1 (C), 128.6 (CH), 121.9 (CH), 120.4 (CH), 119.7 (CH), 109.2 (CH), 98.8 (C), 32.8 (CH_3), 24.6 (CH_3), 20.9 ppm (CH_3). ^{77}Se NMR (76 MHz, $CDCl_3$): δ = 119.5 ppm. MS (EI, 70 eV): *m/z* (%) = 329 (29) $[M]^+$, 197 (21), 131 (100), 105 (27), 77 (14), 44 (21).

3-Benzylselanyl-1-methyl-1*H*-indole (**3aj**) [2]

Yield: 123 mg (82%); Orange oil (from CH_2Cl_2-Hexane); R_f = 0.21 (CH_2Cl_2-Hexane, 1:5). ^{1}H NMR (400 MHz, $CDCl_3$): δ = 7.64 (dd, J = 7.8, 0.9 Hz, 1H; Ar-H), 7.33 (d, J = 8.2 Hz, 1H; Ar-H), 7.29–7.24 (m, *2*H; Ar-H), 7.19–7.14 (m, 3H; Ar-H), 7.05 (dt, J = 7.8, 1.8 Hz, 2H; Ar-H), 6.92 (s, 1H; Ar-H), 3.86 (s, 2H; CH_2), 3.76 ppm (s, 3H; *N*-CH_3). ^{13}C NMR (100 MHz, $CDCl_3$): δ = 139.8 (C), 137.2 (C), 135.1 (CH), 130.8 (C), 128.8 (CH), 128.1 (CH), 126.4 (CH), 122.1 (CH), 120.2 (CH), 120.0 (CH), 109.3 (CH), 96.9 (C), 32.8 (CH_3), 32.5 ppm (CH_2). ^{77}Se NMR (76 MHz, $CDCl_3$): δ = 189.6 ppm. MS (EI, 70 eV): *m/z* (%) = 301 (39) $[M]^+$, 210 (100), 130 (30), 91 (28).

5-Methoxy-1-methyl-3-phenylselanyl-1*H*-indole (**3ba**) [3]

Yield: 140 mg (89%); Colorless plate (from CH_2Cl_2-Hexane); m.p. 93.0–93.5 °C; R_f = 0.77 (Et_2O-Hexane, 1:10). ^{1}H NMR (400 MHz, $CDCl_3$): δ = 7.29 (s, 1H; Ar-H), 7.26 (s, 1H; Ar-H), 7.22 (dt, J = 6.4, 1.8 Hz, 2H; Ar-H), 7.15–7.08 (m, 3H; Ar-H), 7.05 (d, J = 2.3 Hz, 1H; Ar-H), 6.93 (dd, J = 8.7, 2.3 Hz, 1H; Ar-H), 3.82 (s, 3H; *O*-CH_3), 3.81 ppm (s, 3H; *N*-CH_3). ^{13}C NMR (100 MHz, $CDCl_3$): δ = 154.9 (C), 136.0 (CH), 134.3 (C), 132.6 (C), 131.4 (C), 128.9 (CH), 128.3 (CH), 125.4 (CH), 113.0 (CH), 110.4 (CH), 101.5 (CH), 95.1 (C), 55.8 (CH_3), 33.2 ppm (CH_3). ^{77}Se NMR (76 MHz, $CDCl_3$): δ = 206.9 ppm. MS (EI, 70 eV): *m/z* (%) = 317 (30) $[M]^+$, 237 (100), 194 (13).

5-Bromo-1-methyl-3-phenylselanyl-1*H*-indole (**3ea**) [4]

Yield: 181 mg (99%); Colorless plate (from CH_2Cl_2-Hexane); m.p. 139.0–141.0 °C; R_f = 0.71 (CH_2Cl_2-Hexane, 1:5). ^{1}H NMR (400 MHz, $CDCl_3$): δ = 7.75 (d, J = 1.8 Hz, 1H; Ar-H), 7.35 (dd, J = 8.7, 1.8 Hz, 1H; Ar-H), 7.31 (s, 1H; Ar-H), 7.24 (d, J = 6.0 Hz, 1H; Ar-H), 7.21–7.19 (m, 2H; Ar-H), 7.16–7.10 (m, 3H; Ar-H), 3.82 ppm (s, 3H; *N*-CH_3). ^{13}C NMR (100 MHz, $CDCl_3$): δ = 136.7 (CH), 136.1 (C), 133.7 (C), 132.4 (C), 129.0 (CH), 128.5 (CH), 125.7 (CH), 125.4 (CH), 122.9 (CH), 114.1 (C), 111.1 (CH), 95.6 (C), 33.2 ppm (CH_3). ^{77}Se NMR (76 MHz, $CDCl_3$): δ = 209.2 ppm. MS (EI, 70 eV): *m/z* (%) = 365 (40) $[M]^+$, 287 (100).

5-Methoxy-3-phenylselanyl-1*H*-indole (**3ja**) [4]

Yield: 140 mg (89%); pale yellow oil; R_f = 0.35 (AcOEt-Hexane, 1:5). ^{1}H NMR (400 MHz, $CDCl_3$): δ = 8.35 (brs, 1H; NH), 7.37 (d, J = 2.3 Hz, 1H; Ar-H), 7.26 (d, J = 8.7 Hz, 1H; Ar-H), 7.23–7.20 (m, 2H; Ar-H), 7.14–7.05 (m, 4H; Ar-H), 6.03 (dd, J = 8.7, 2.3 Hz, 1H; Ar-H), 3.77 ppm (s, 3H; *O*-CH_3). ^{13}C NMR (100 MHz, $CDCl_3$): δ = 155.0 (C), 133.9 (C), 131.9 (CH), 131.2 (C), 130.7 (C), 128.9 (CH), 128.4 (CH), 125.5 (CH), 113.4 (CH), 112.2 (CH), 101.4 (CH), 97.4 (C), 55.7 ppm (CH_3). ^{77}Se NMR (76 MHz, $CDCl_3$): δ = 210.4 ppm. MS (EI, 70 eV): *m/z* (%) = 303 (40) $[M]^+$, 223 (100), 180 (13).

3-Phenylselanyl-1*H*-indole (**3ka**) [1]

Yield: 126 mg (93%); Colorless prisms (from CH_2Cl_2-Hexane); m.p. 147.0–148.0 °C; R_f = 0.20 (CH_2Cl_2-Hexane, 1:2). ^{1}H NMR (400 MHz, $CDCl_3$): δ = 8.41 (s, 1H; Ar-H), 7.63 (d, J = 7.8 Hz, 1H; Ar-H), 7.48 (d, J = 2.7 Hz, 1H; Ar-H), 7.44 (d, J = 8.2 Hz, 1H; Ar-H), 7.29–7.06 ppm (m, 7H; Ar-H). ^{13}C NMR (100 MHz, $CDCl_3$): δ = 136.4 (C), 133.8 (C), 131.2(CH), 129.9 (C), 128.9 (CH), 128.6 (CH), 125.6 (CH), 122.9 (CH), 120.9 (CH), 120.4 (CH), 111.3 (CH), 98.1 ppm (C). ^{77}Se NMR (76 MHz, $CDCl_3$): δ = 211.6 ppm. MS (EI, 70 eV): m/z (%) = 273 (20) $[M]^+$, 193 (100), 165 (15), 117 (15), 89 (15).

3-phenylselanyl-1*H*-indole-5-carbonitrile (**3la**) [5]

Yield: 117 mg (79%); pale yellow oil; R_f = 0.30 (AcOEt-Hexane, 2:3). ^{1}H NMR (400 MHz, $CDCl_3$): δ = 8.84 (brs, 1H; NH), 7.98 (s, 1H; Ar-H), 7.62 (d, J = 2.3 Hz, 1H; Ar-H), 7.51 (d, J = 7.7 Hz, 1H; Ar-H), 7.48 (dd, J = 8.2, 1.8 Hz, 1H; Ar-H), 7.24–7.14 ppm (m, 5H; Ar-H). ^{13}C NMR (100 MHz, $CDCl_3$): δ = 138.2 (C), 133.1 (CH), 132.5 (C), 129.9 (C), 129.2 (CH), 129.1 (CH), 126.2 (CH), 126.1 (CH), 125.9 (CH), 120.3 (C), 112.4 (CH), 104.1 (C), 99.7 ppm (C). ^{77}Se NMR (76 MHz, $CDCl_3$): δ = 215.4 ppm. MS (EI, 70 eV): m/z (%) = 289 (20) $[M]^+$, 218 (100), 190 (15).

1-Bnzyl-3-phenylselanyl-1*H*-indole (**3ma**) [1]

Yield: 168 mg (93%); Colorless prism (from CH_2Cl_2-Hexane); m.p. 79.0–81.0 °C; R_f = 0.46 (CH_2Cl_2-Hexane, 1:5). ^{1}H NMR (400 MHz, $CDCl_3$): δ = 7.64 (d, J = 7.8 Hz, 1H; Ar-H), 7.40 (s, 1H; Ar-H), 7.34–7.26 (m, 4H; Ar-H), 7.25–7.21 (m, 3H; Ar-H), 7.18–7.08 (m, 6H; Ar-H), 5.36 ppm (s, 2H; *N*-CH_2). ^{13}C NMR (100 MHz, $CDCl_3$): δ = 137.2 (C), 136.8 (C), 135.1 (CH), 134.2 (C), 131.0 (C), 129.04 (CH), 129.01 (CH), 128.6 (CH), 128.0 (CH), 127.1 (CH), 125.6 (CH), 122.7 (CH), 120.76 (CH), 120.73 (CH), 110.2 (CH), 96.9 (C), 50.5 ppm (CH_2). ^{77}Se NMR (76 MHz, $CDCl_3$): δ = 210.8 ppm. MS (EI, 70 eV): m/z (%) = 363 (28) $[M]^+$, 283 (89), 192 (44), 165 (22), 91 (100), 65 (17).

1-Phenyl-3-phenylselanyl-1*H*-indole (**3na**) [4]

Yield: 174 mg (99%); Yellow oil (from CH_2Cl_2-Hexane); R_f = 0.34 (CH_2Cl_2-Hexane, 1:5). ^{1}H NMR (400 MHz,

$CDCl_3$): δ = 7.68 (d, J = 7.8 Hz, 1H; Ar-H), 7.62 (s, 1H; Ar-H), 7.6 (dd, J = 8.2, 0.9 Hz, 1H; Ar-H), 7.55–7.52 (m, 4H; Ar-H), 7.41–7.37 (m, 1H; Ar-H), 7.32–7.09 ppm (m, 7H; Ar-H). ^{13}C NMR (100 MHz, $CDCl_3$): δ = 139.0 (C), 136.7 (C), 134.4 (CH), 133.5 (C), 131.2 (C), 129.7 (CH), 129.0 (CH), 128.9 (CH), 127.0 (CH), 125.7 (CH), 124.4 (CH), 123.2 (CH), 121.3 (CH), 120.8 (CH), 110.8 (CH), 99.3 ppm (C). ^{77}Se NMR (76 MHz, $CDCl_3$): δ = 213.6 ppm. MS (EI, 70 eV): m/z (%) = 349 (17) $[M]^+$, 269 (100), 165 (33).

1-Methyl-2-phenyl-3-phenylselanyl-1*H*-indole (**3pa**) [6]

Yield: 180 mg (99%); Colorless prisms (from CH_2Cl_2-Hexane); m.p. 104.0–105.0 °C; R_f = 0.20 (CH_2Cl_2-Hexane, 1:5). ^{1}H NMR (400 MHz, $CDCl_3$): δ = 7.66 (d, J = 0.9 Hz, 1H; Ar-H), 7.44–7.37 (m, 6H; Ar-H), 7.32 (td, J = 6.9, 0.9 Hz, 1H; Ar-H), 7.23–7.18 (m, 1H; Ar-H), 7.17–7.14 (m, 2H; Ar-H), 7.12–7.05 (m, 3H; Ar-H), 3.73 ppm (s, 3H; *N*-CH_3). ^{13}C NMR (100 MHz, $CDCl_3$): δ = 145.9 (C), 137.7 (C), 134.6 (C), 131.2 (C), 130.8 (CH), 130.6 (C), 128.8 (CH), 128.7 (CH), 128.3 (CH), 128.1 (CH), 125.2 (CH), 122.7 (CH), 120.8 (CH), 120.6 (CH), 109.7 (CH), 96.3 (C), 31.8 ppm (CH_3). ^{77}Se NMR (76 MHz, $CDCl_3$): δ = 209.2 ppm. MS (EI, 70 eV): m/z (%) = 363 (20) $[M]^+$, 283 (100), 207 (80), 78 (25), 44 (25).

1,2-Dimethyl-3-phenylselanyl-1*H*-indole (**3qa**) [1]

Yield: 137 mg (91%); Colorless plate (from CH_2Cl_2-Hexane); m.p. 124.0–125.5 °C; R_f = 0.28 (CH_2Cl_2-Hexane, 1:5). ^{1}H NMR (400 MHz, $CDCl_3$): δ = 7.58 (d, J = 7.8 Hz, 1H; Ar-H), 7.31 (d, J = 8.2 Hz, 1H; Ar-H), 7.22 (td, J = 6.9, 1.4 Hz, 1H; Ar-H), 7.16–7.03 (m, 6H; Ar-H), 3.76 (s, 3H; *N*-CH_3), 2.56 ppm (s, 3H; CH_3). ^{13}C NMR (100 MHz, $CDCl_3$): δ = 142.6 (C), 137.4 (C), 134.4 (C), 130.7 (C), 129.0 (CH), 128.3 (CH), 125.3 (CH), 121.7 (CH), 120.5 (CH), 119.9 (CH), 109.0 (CH), 95.2 (C), 30.6 (CH_3), 12.1 ppm (CH_3). ^{77}Se NMR (76 MHz, $CDCl_3$): δ = 189.7 ppm. MS (EI, 70 eV): m/z (%) = 301 (21) $[M]^+$, 221 (100), 144 (43).

1-Methyl-3-phenylthio-1*H*-indole (**4**) [1]

Yield: 119 mg (99%); Colorless prisms (from CH_2Cl_2-Hexane); m.p. 89.0-91.0 °C; R_f = 0.30 (CH_2Cl_2-Hexane, 1:3).

^{1}H NMR (400 MHz, $CDCl_3$): δ = 7.61 (d, J = 7.8 Hz, 1H; Ar-H), 7.38 (d, J = 8.2 Hz, 1H; Ar-H), 7.33 (s, 1H; Ar-H), 7.29 (t, J = 6.9 Hz, 1H; Ar-H), 7.18–7.12 (m, 3H; Ar-H), 7.10–7.08 (m, 2H; Ar-H), 7.03 (t, J = 7.3 Hz, 1H; Ar-H), 3.84 ppm (s, 3H; *N*-CH_3). ^{13}C NMR (100 MHz, $CDCl_3$): δ = 139.6 (C), 137.5 (C), 135.1(CH), 129.8 (C), 128.6 (CH), 125.7 (CH), 124.6 (CH), 122.5 (CH), 120.5 (CH), 120.0 (CH), 109.7 (CH), 100.4 (C), 33.1 ppm (CH_3). MS (EI, 70 eV): *m/z* (%) = 239 (100) $[M]^+$, 223 (20), 207 (20), 162 (20), 77 (20).

1-Methyl-3-phenyltelluro-1*H*-indole (**5**) [7]

Yield: 43 mg (26%); Colorless prisms (from CH_2Cl_2-Hexane); m.p. 157.0-158.5 °C; R_f = 0.30 (CH_2Cl_2-Hexane, 1:5). ^{1}H NMR (400 MHz, $CDCl_3$): δ = 7.61 (d, J = 7.8 Hz, 1H; Ar-H), 7.41–7.38 (m, 3H; Ar-H), 7.35 (d, J = 8.2 Hz, 1H; Ar-H), 7.28 (td, J = 6.9, 0.9 Hz, 1H; Ar-H), 7.19 (td, J = 8.2, 1.4 Hz, 1H; Ar-H), 7.12–7.02 (m, 3H; Ar-H), 3.82 ppm (s, 3H; *N*-CH_3). ^{13}C NMR (100 MHz, $CDCl_3$): δ = 139.9 (CH), 137.5 (C), 134.2(CH), 133.2 (C), 129.1 (CH), 126.5 (CH), 122.4 (CH), 122.2 (CH), 120.4 (CH), 116.8 (C), 109.4 (CH), 78.8 (C), 33.0 ppm (CH_3). MS (EI, 70 eV): *m/z* (%) = 337 (15) $[M]^+$, 207 (100), 165 (10), 130 (30), 77 (20).

2. References

1. Rios, E.A.M.; Gomes, C.M.B.; Silvério, G.L.; Luz, E.Q.; Ali, S.; D'Oca, C.d.R.M.; Albach, B.; Campos, R.B.; Rampon, D.S. *RSC Adv*. **2023**, *13*, 914–925.
2. Guo, T.; Dong, Z.; Zhang, P.; Xing, W.; Li, L. *Tetrahedron Lett.* **2018**, *59*, 2554–2558.
3. Benchawan, T.; Maneewong, J.; Saeeng, R. *ChemistrySelect* **2023**, *8*, e202301988.
4. Zhang, X.; Wang, C.; Jiang, H.; Sun, L. *Chem. Commun*. **2018**, *54*, 8781–8784.
5. Zhang, Q.-B.; Ban, Y.-L.; Yuan, P.-F.; Peng, S.-J.; Fang, J.-G.; Wu, L.-Z.; Liu, Q. *Green Chem*. **2017**, *19*, 5559–5563.
6. Lin, M.; Kang, L.; Gu, J.; Dai, L.; Tang, S.; Zhang, T.; Wang, Y.; Li, L.; Zheng, X.; Zhu, W.; Si, R.; Fu, X.; Sun, L.; Zhang, Y.; Yan, C. *Nano Res.* **2017**, *10*, 922–932.
7. Chen, J.; Hu, L.; Wang, H.; Tan, H. *Chin. J. Org. Chem.* **2019,** *39,* 2048–2052.

3. Copies of ^{1}H and ^{13}C NMR spectra of products

^{1}H NMR of **3aa**

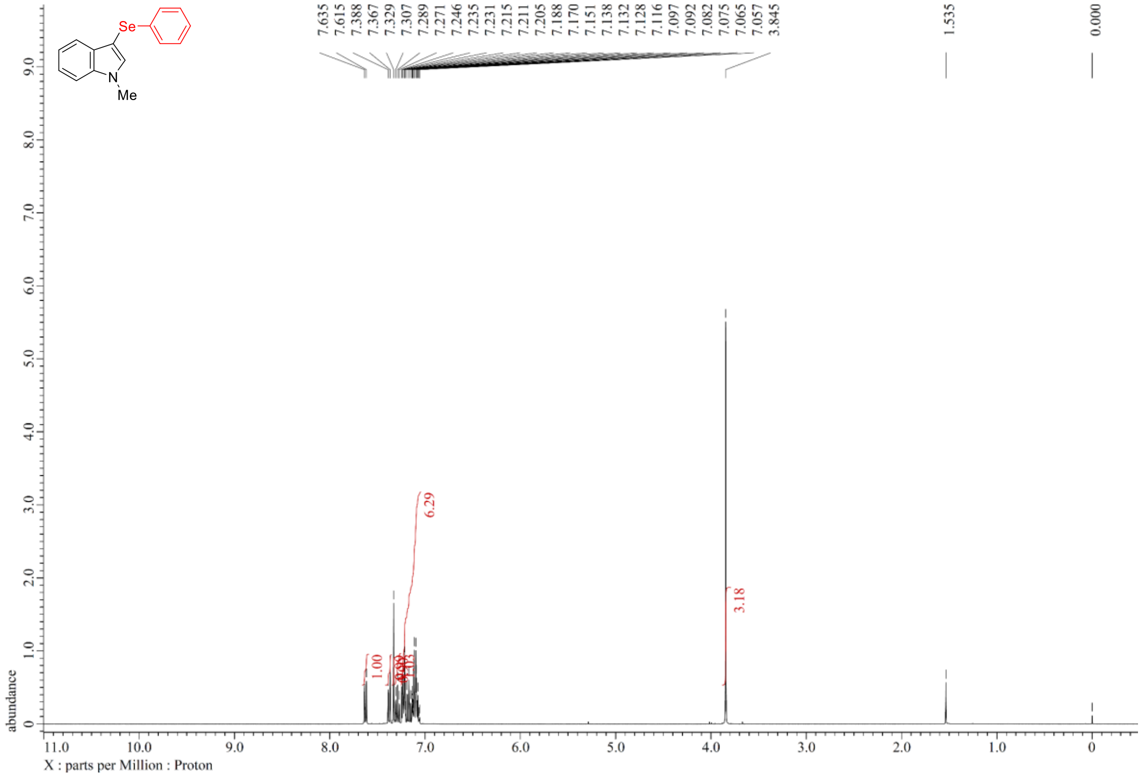

^{13}C NMR of **3aa**

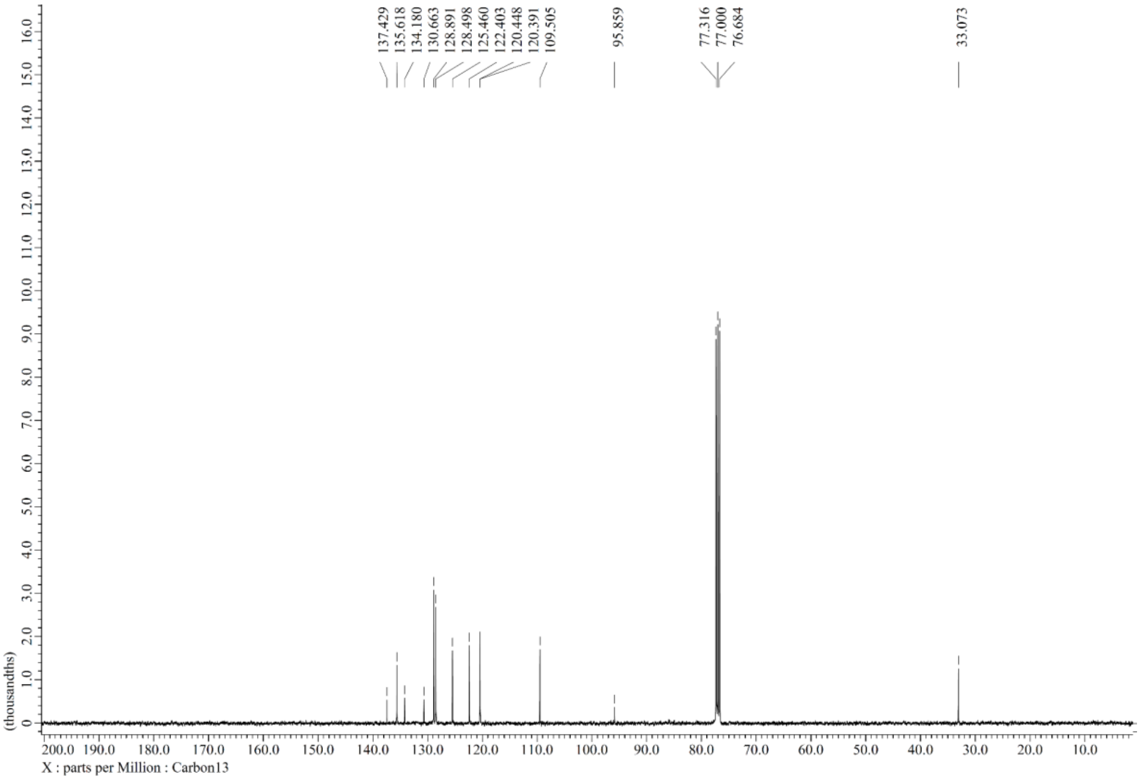

^{1}H NMR of **3ab**

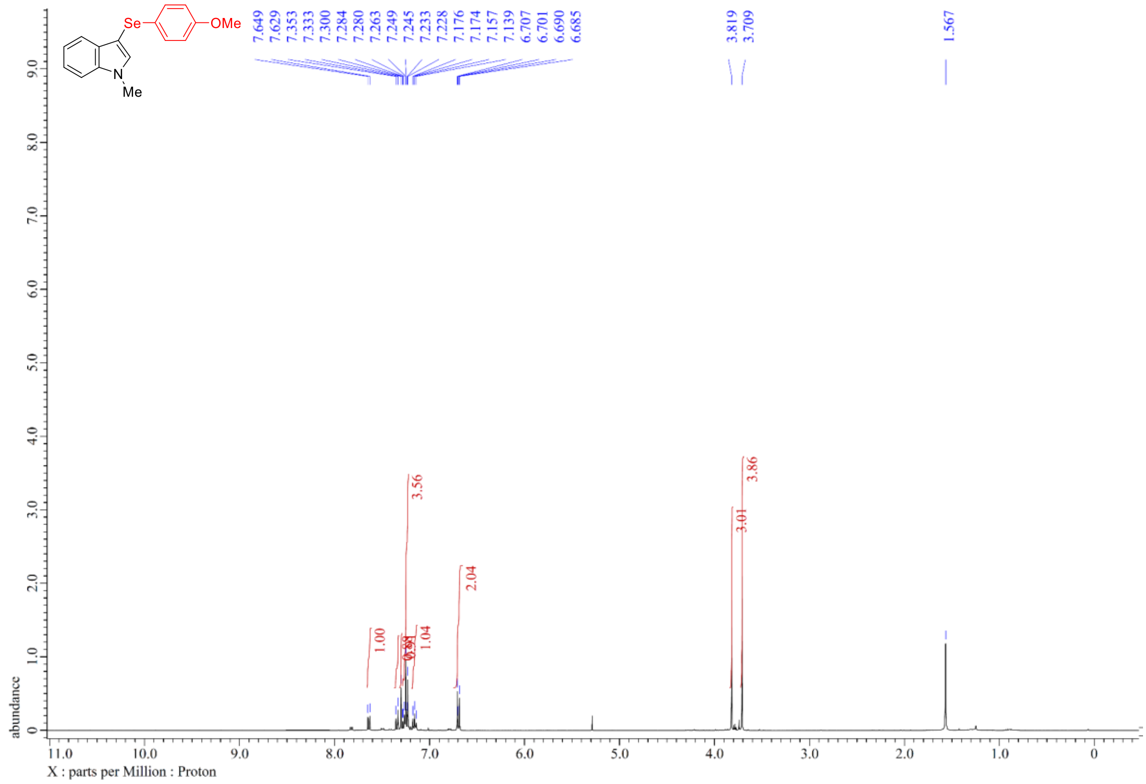

^{13}C NMR of **3ab**

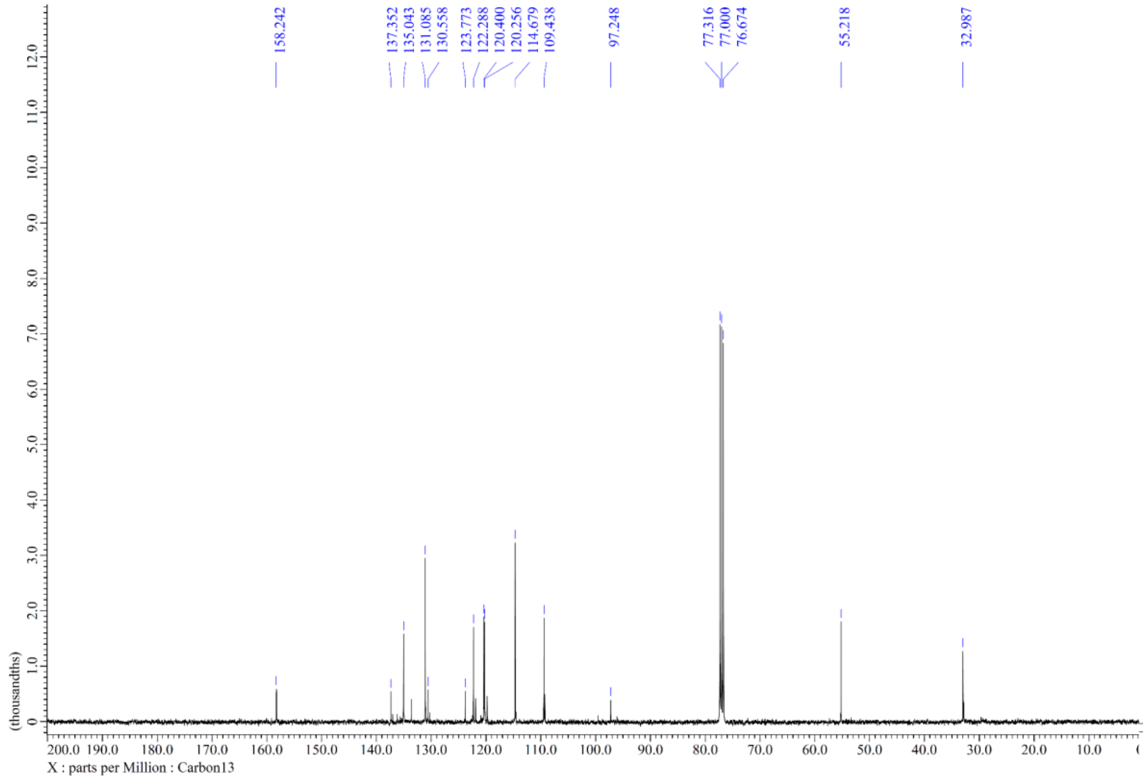

^{1}H NMR of **3ac**

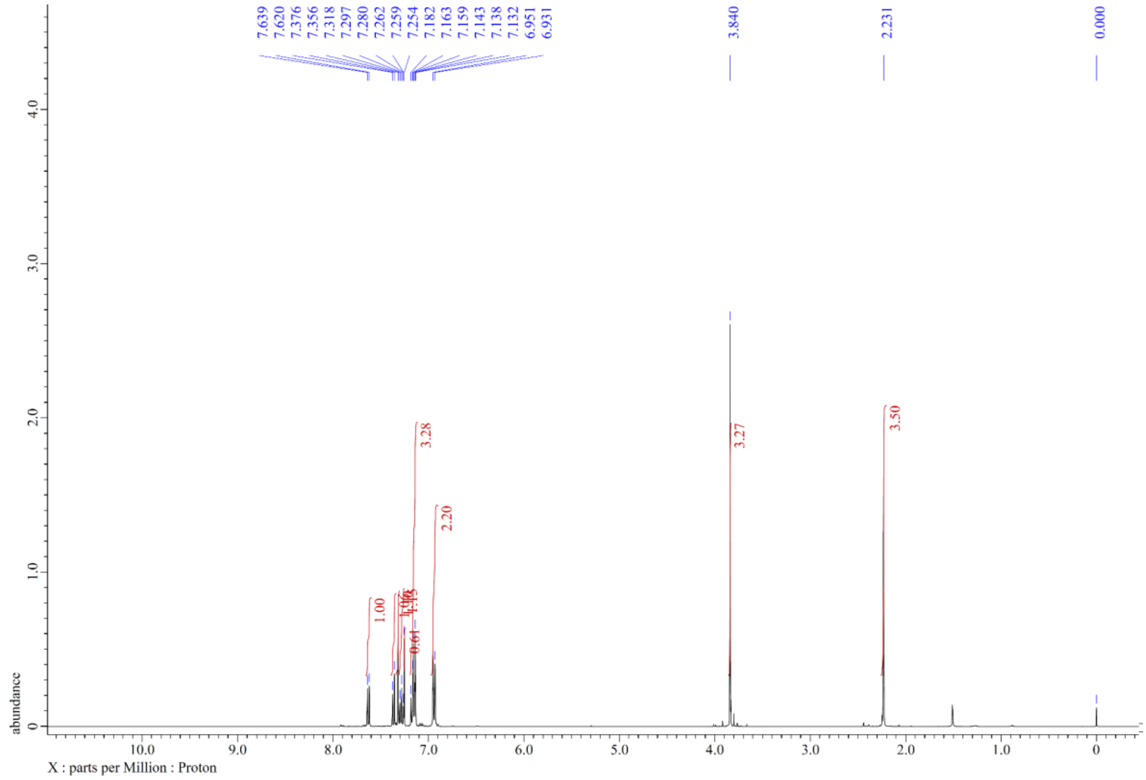

^{13}C NMR of **3ac**

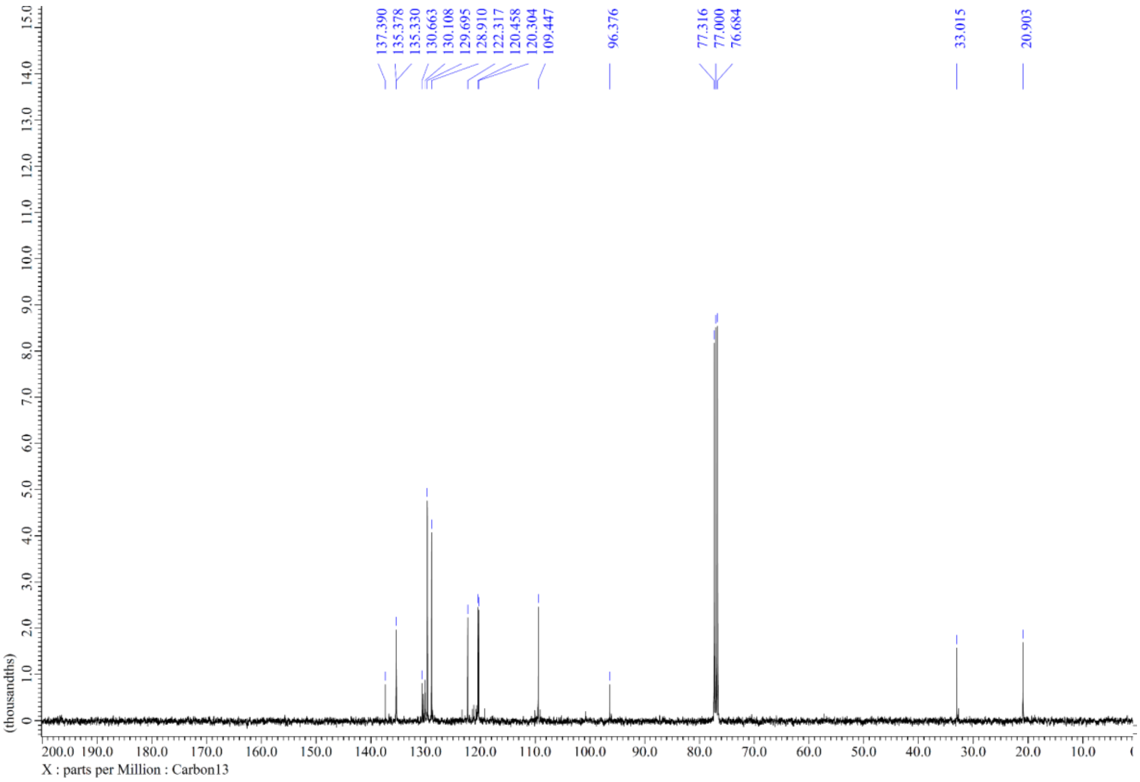

^{1}H NMR of **3ad**

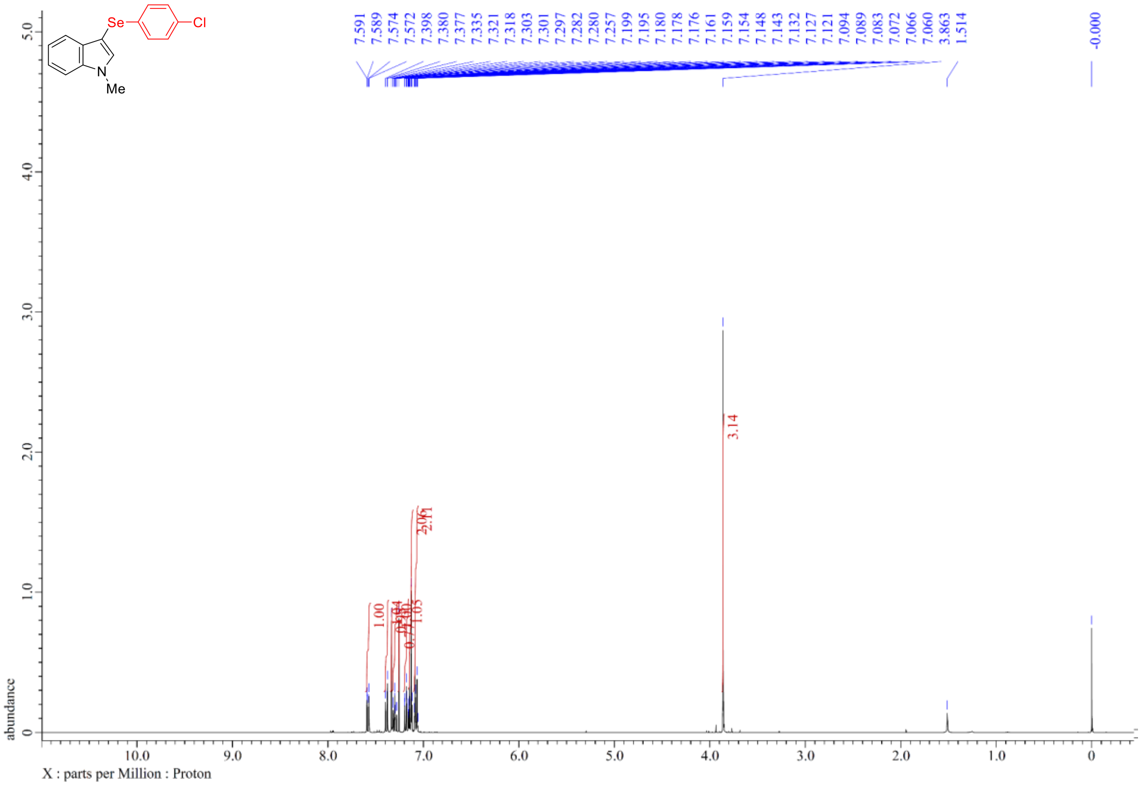

^{13}C NMR of **3ad**

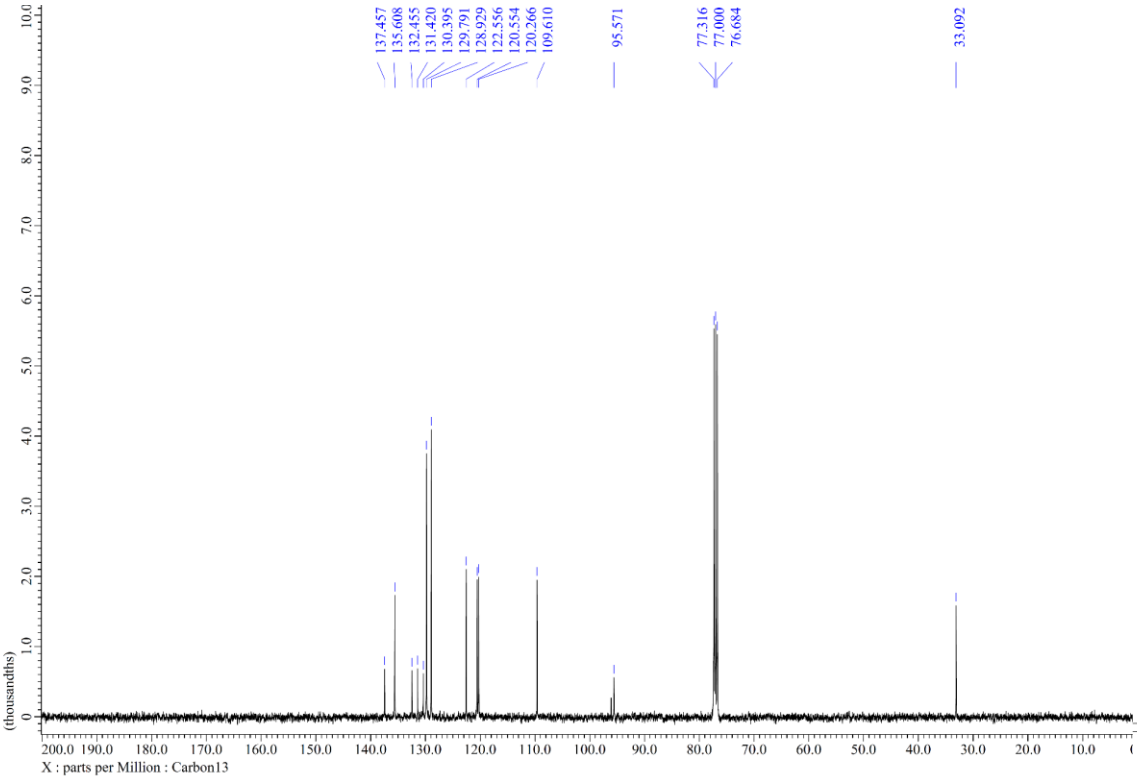

^{1}H NMR of **3ae**

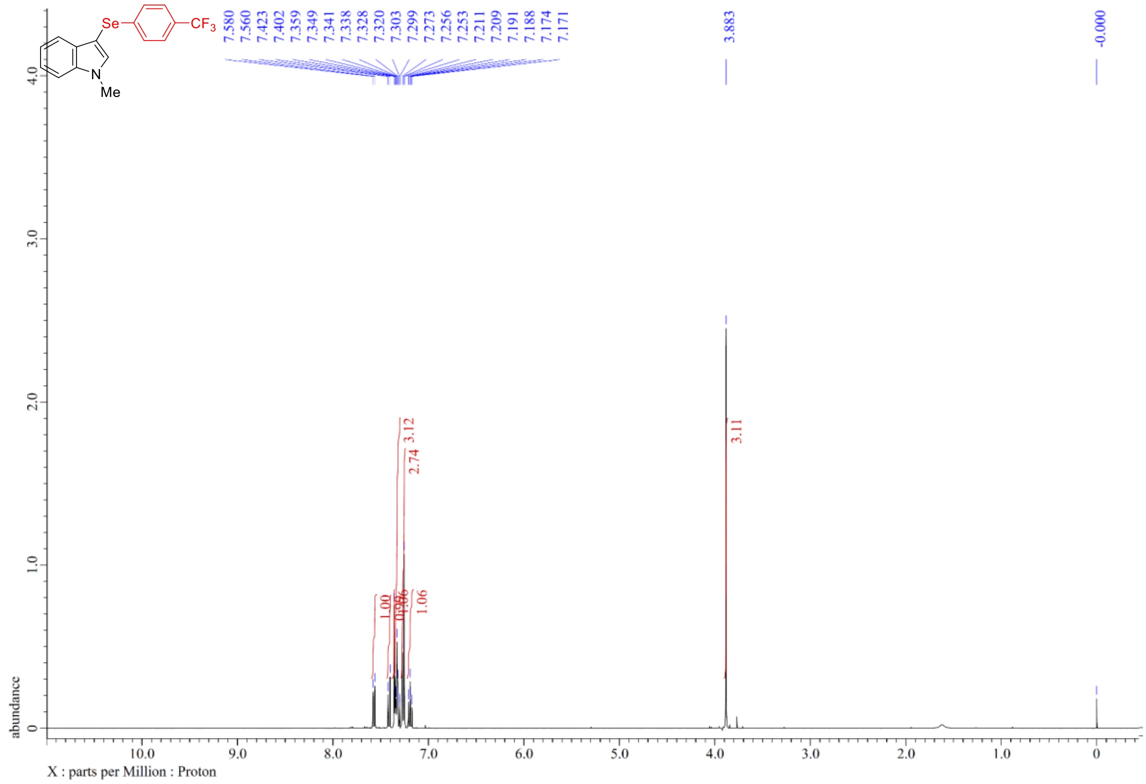

^{13}C NMR of **3ae**

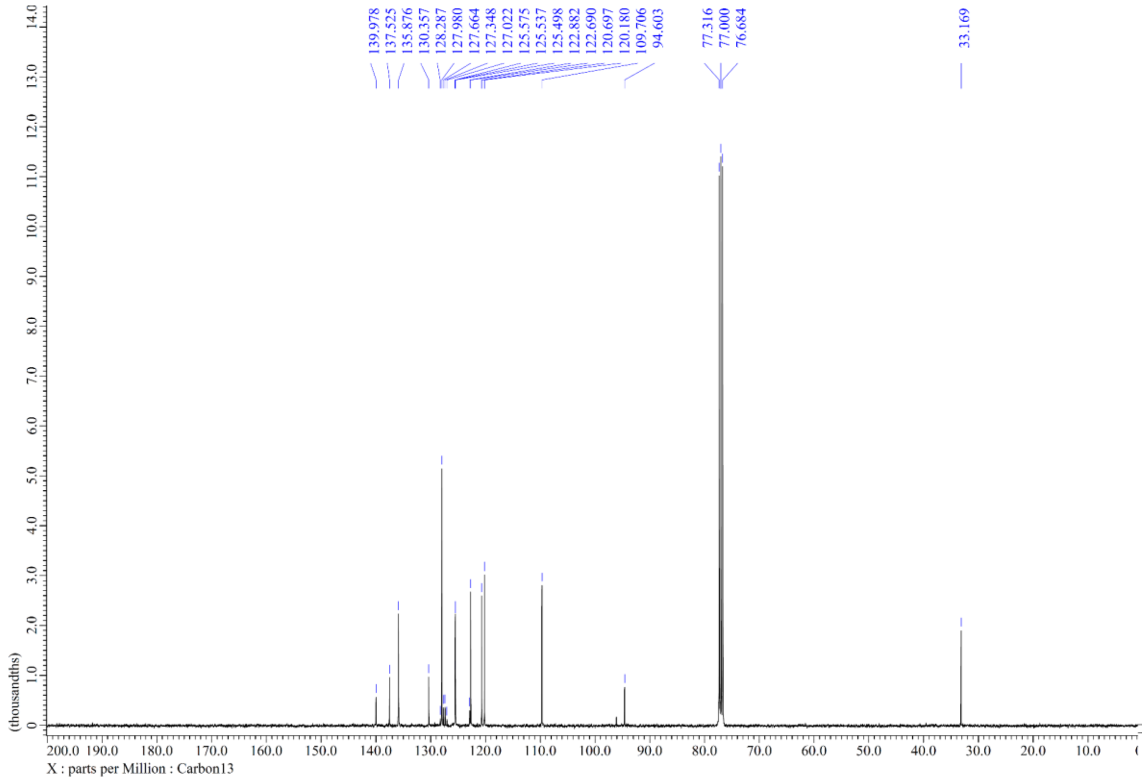

^{1}H NMR of **3af**

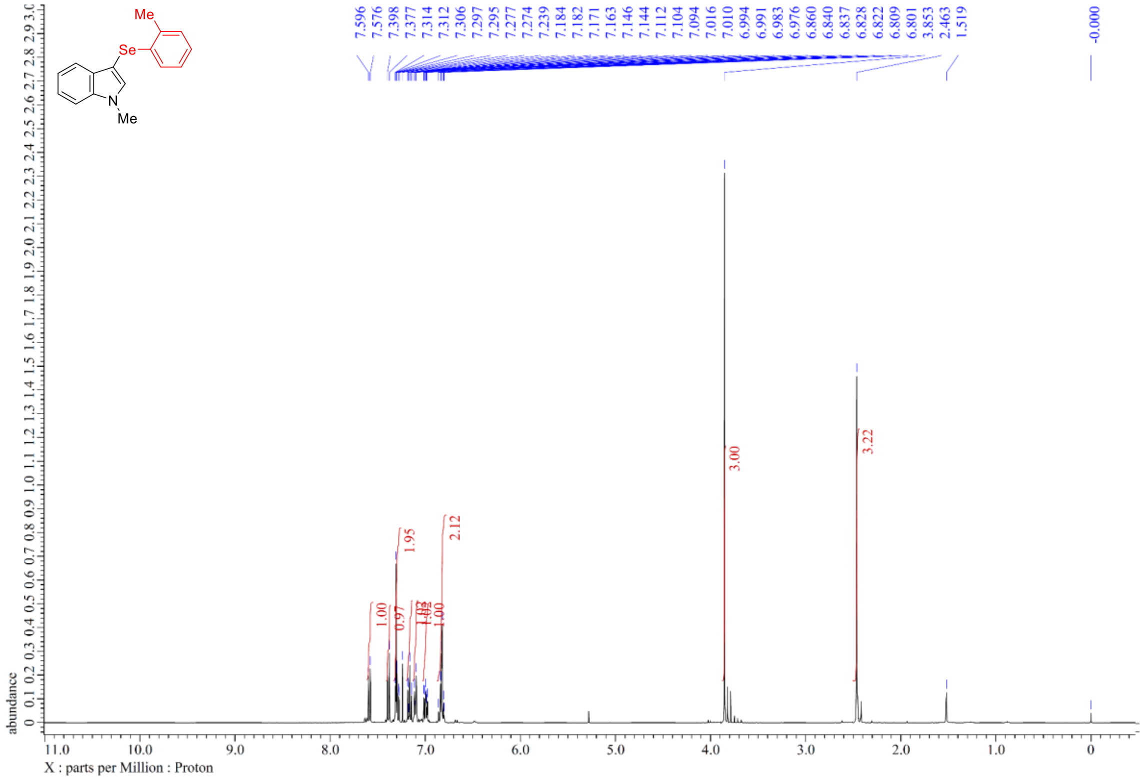

^{13}C NMR of **3af**

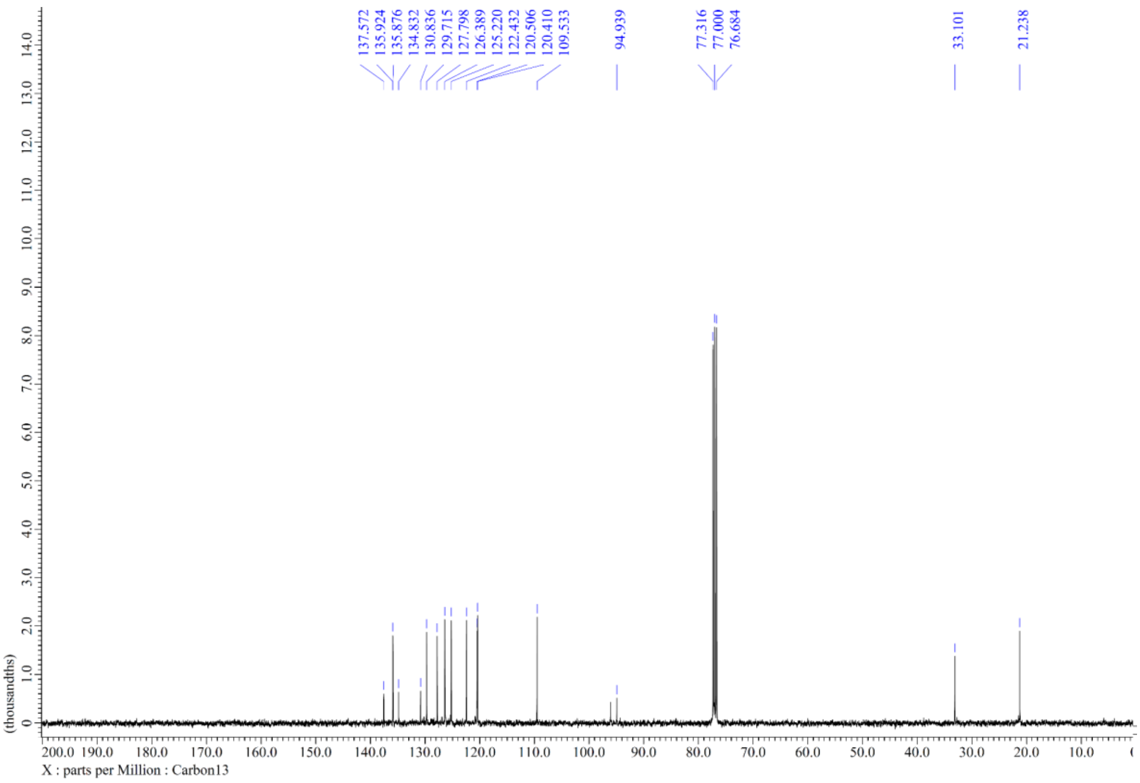

^{1}H NMR of **3ag**

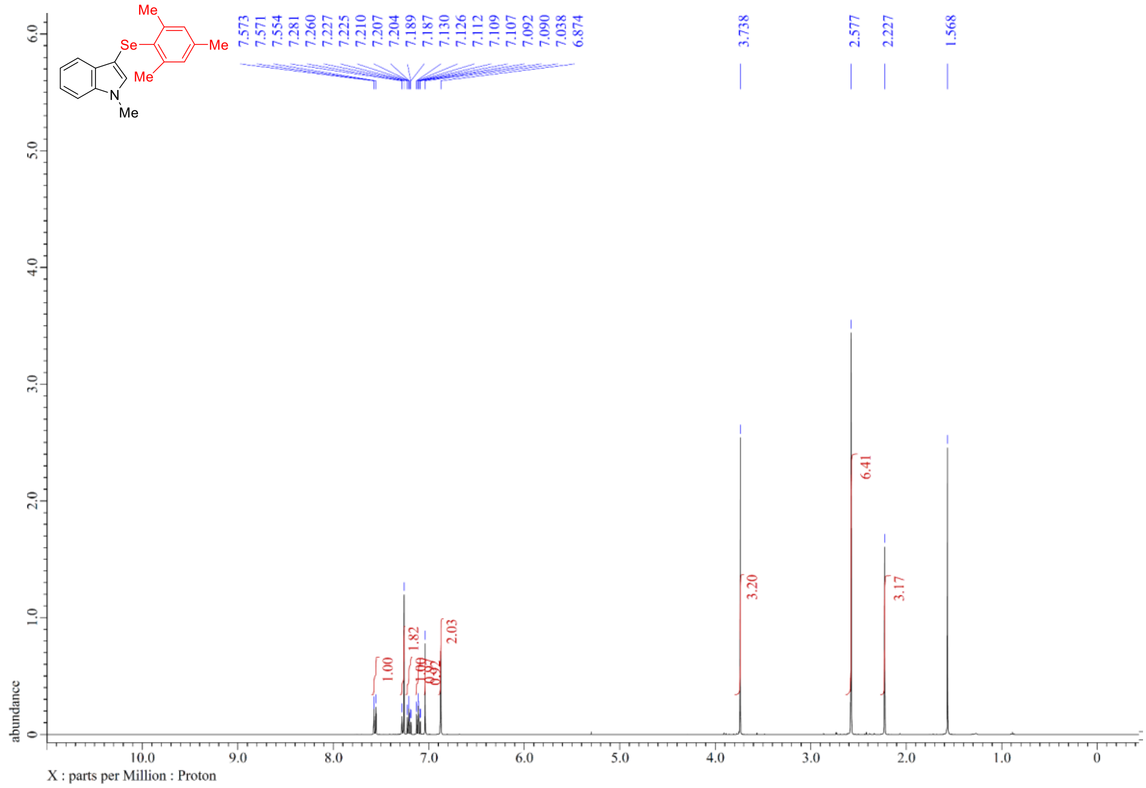

^{13}C NMR of **3ag**

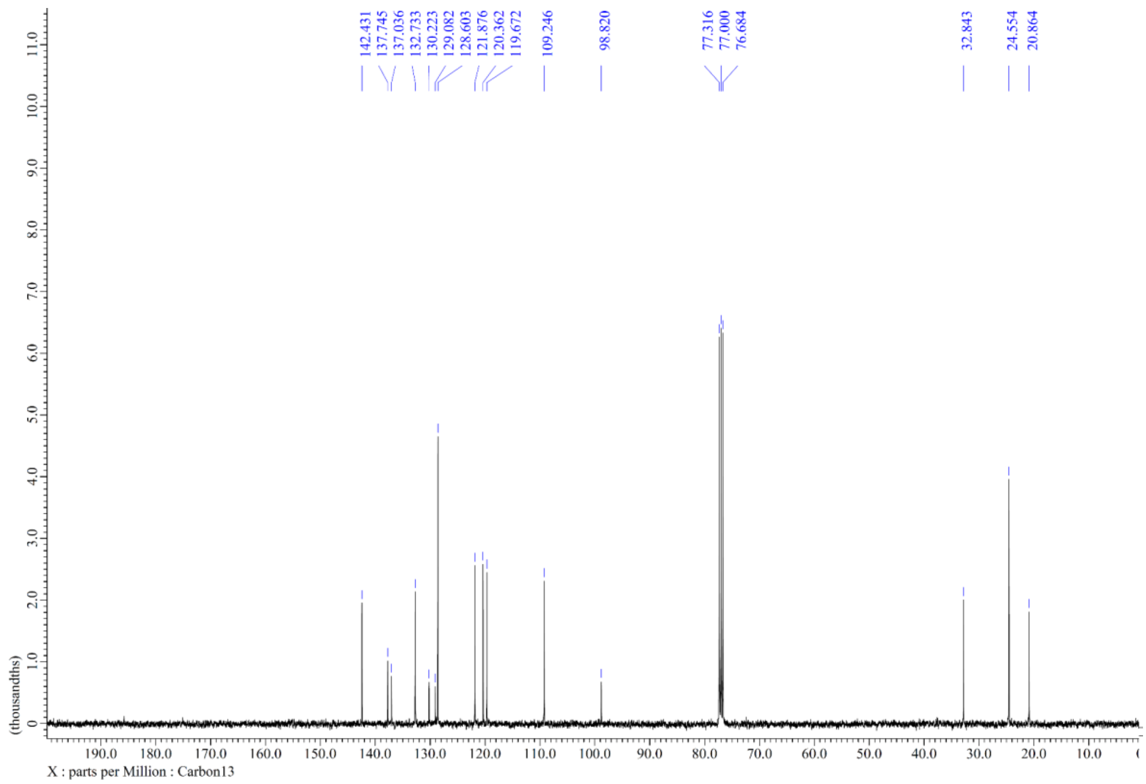

^{1}H NMR of **3ai**

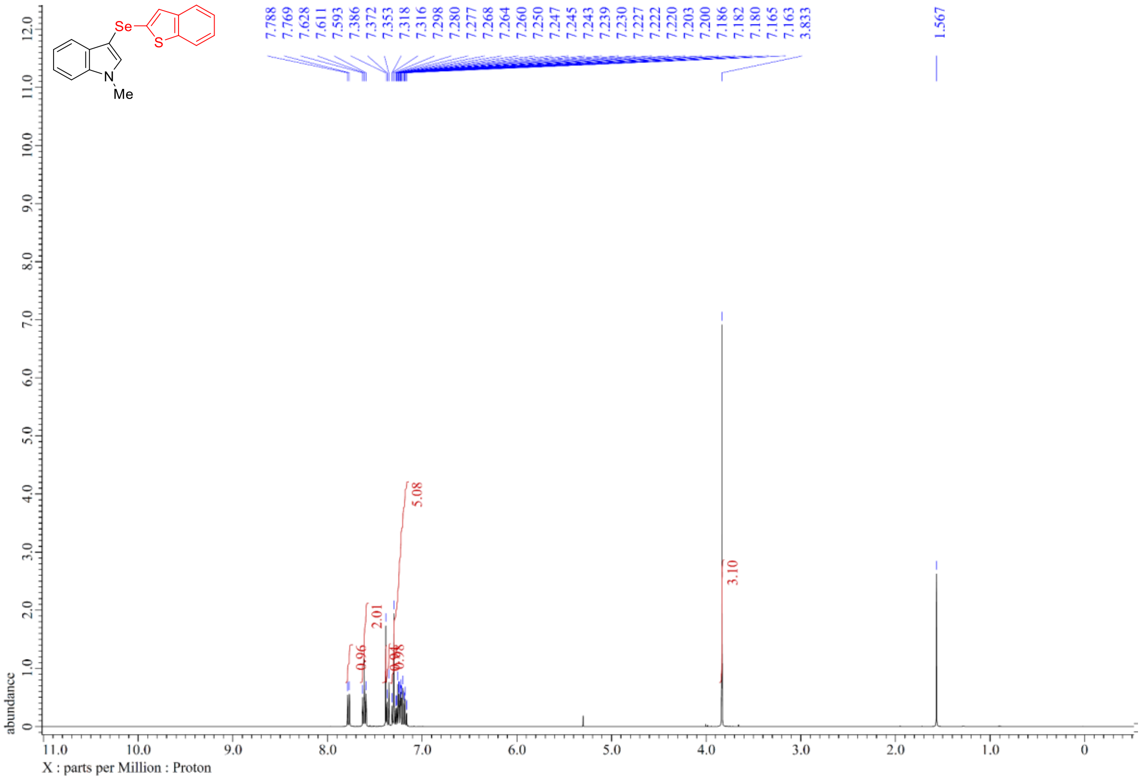

^{13}C NMR of **3ai**

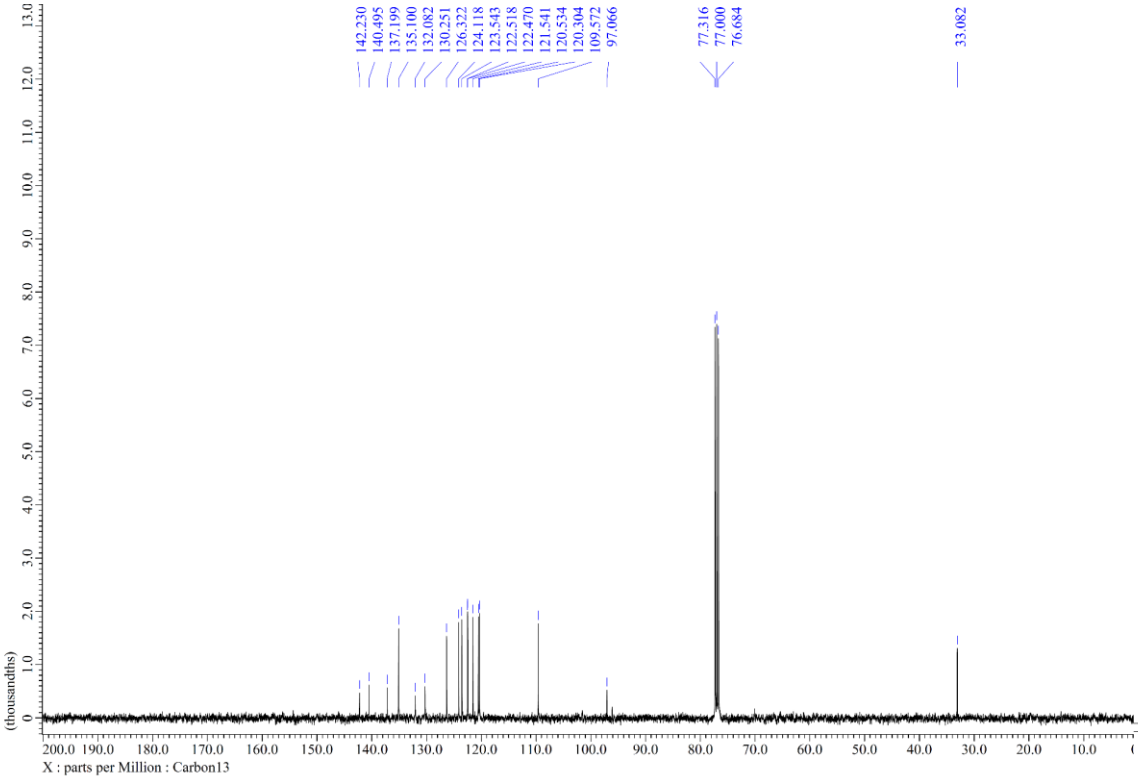

^{1}H NMR of **3aj**

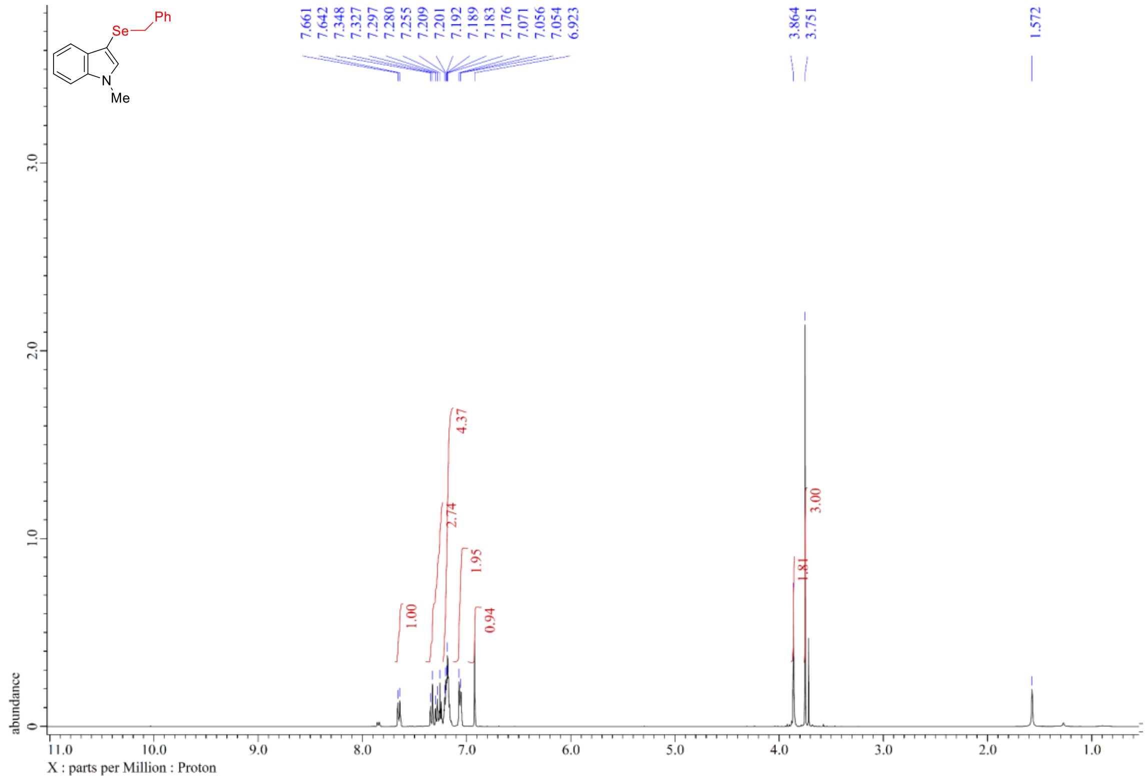

^{13}C NMR of **3aj**

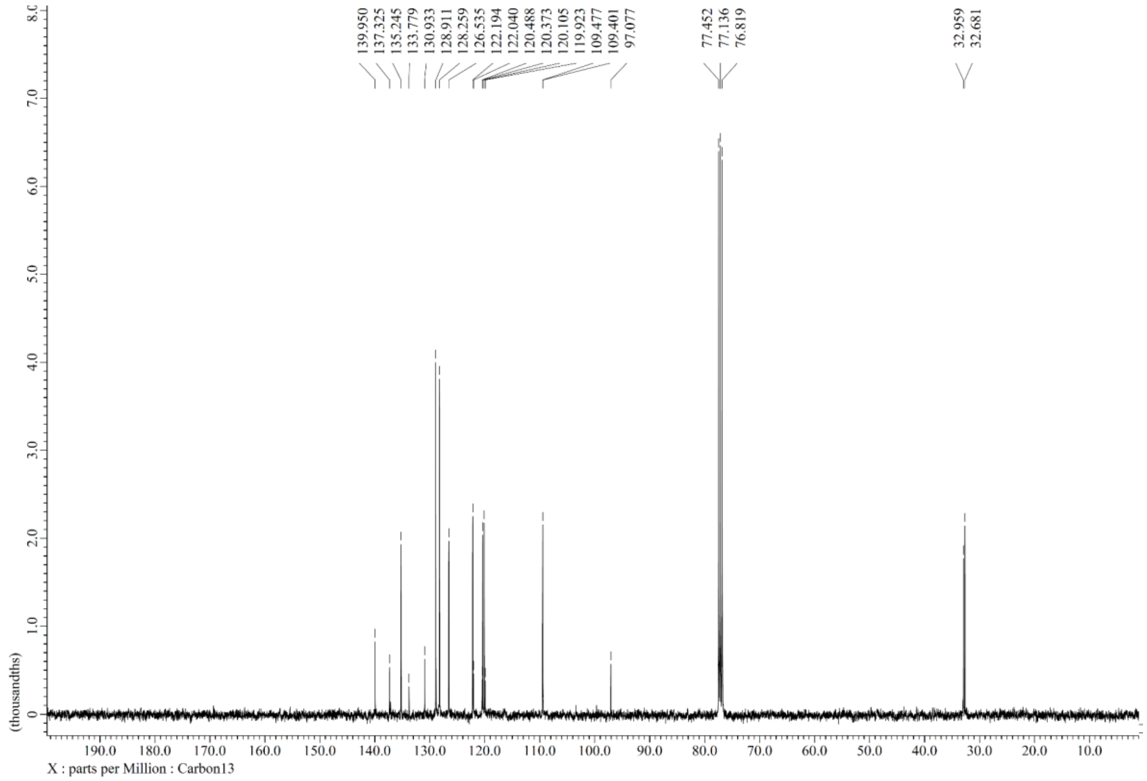

^{1}H NMR of **3ba**

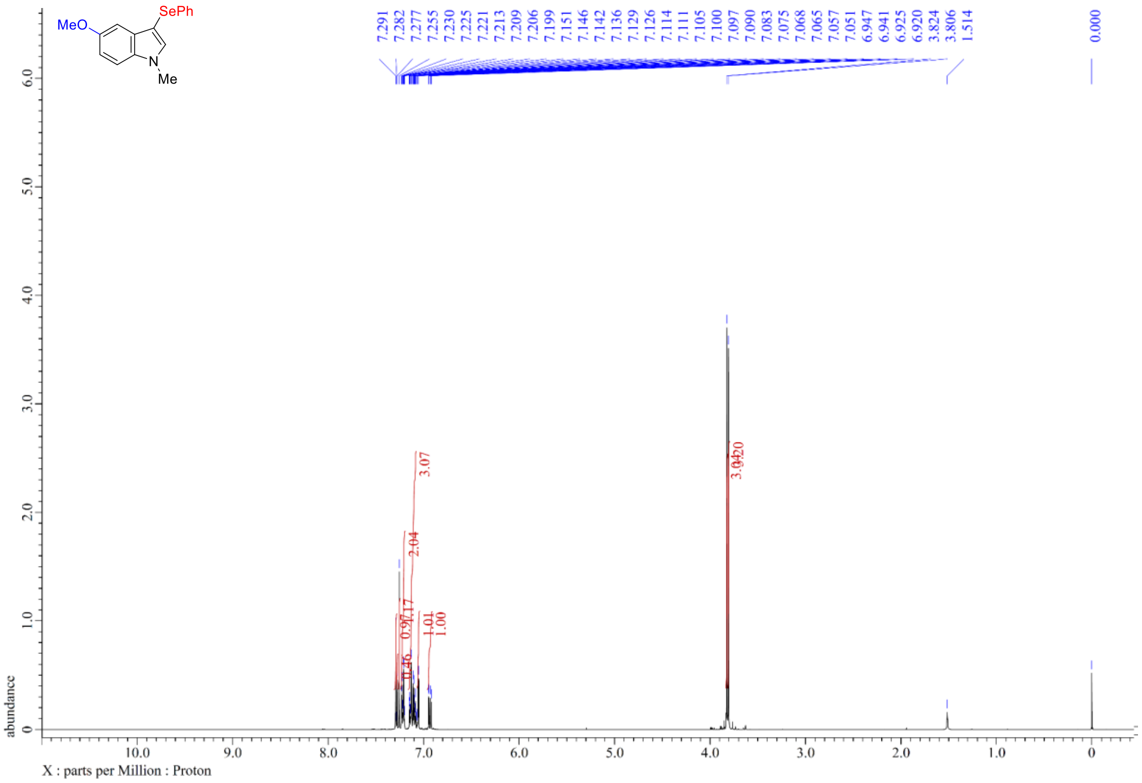

^{13}C NMR of **3ba**

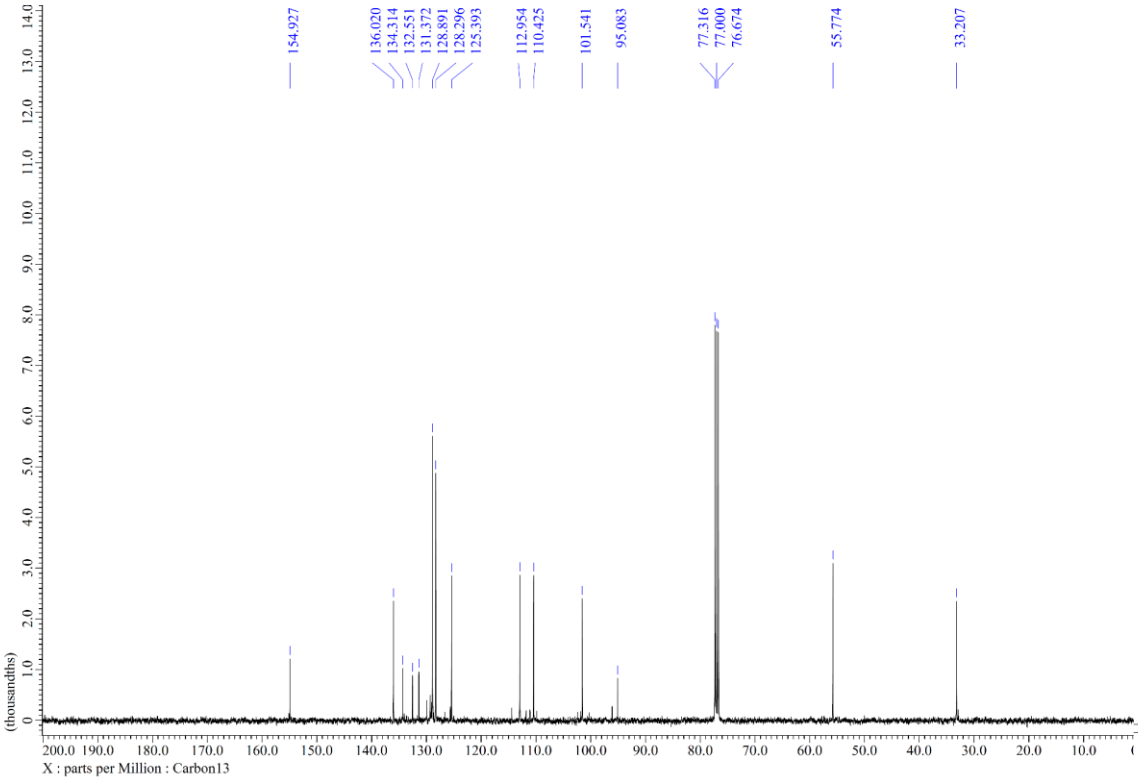

^{1}H NMR of **3ca**

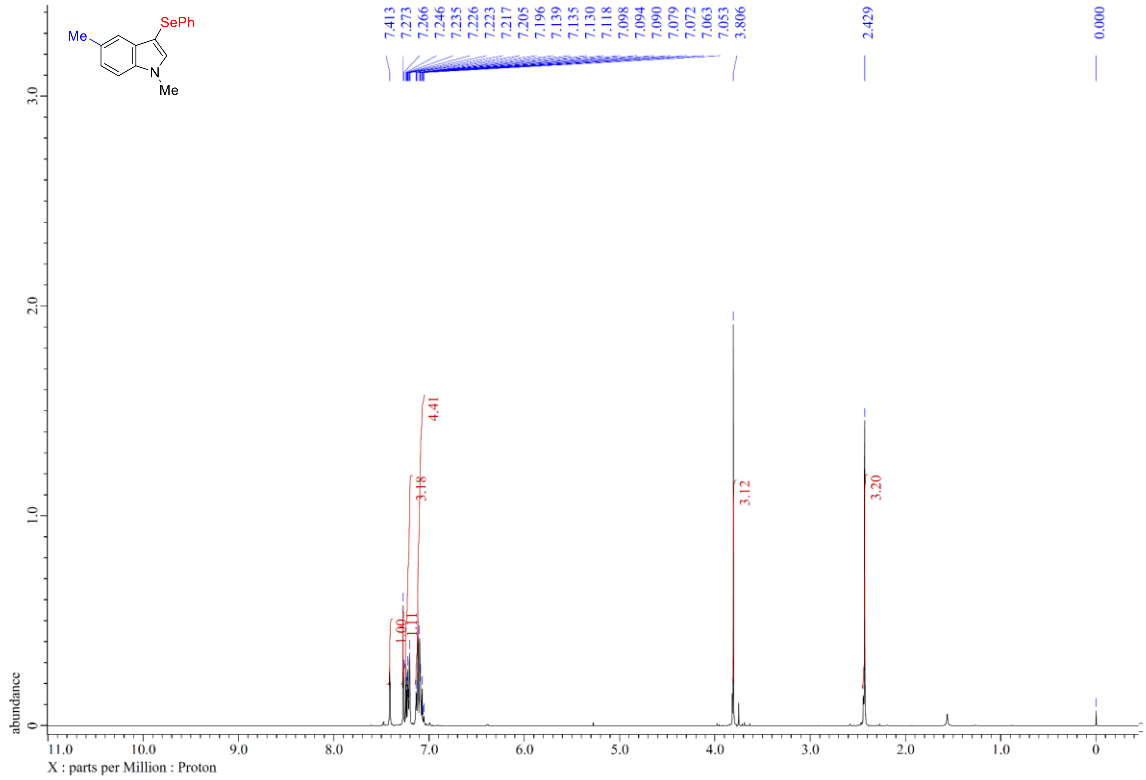

^{13}C NMR of **3ca**

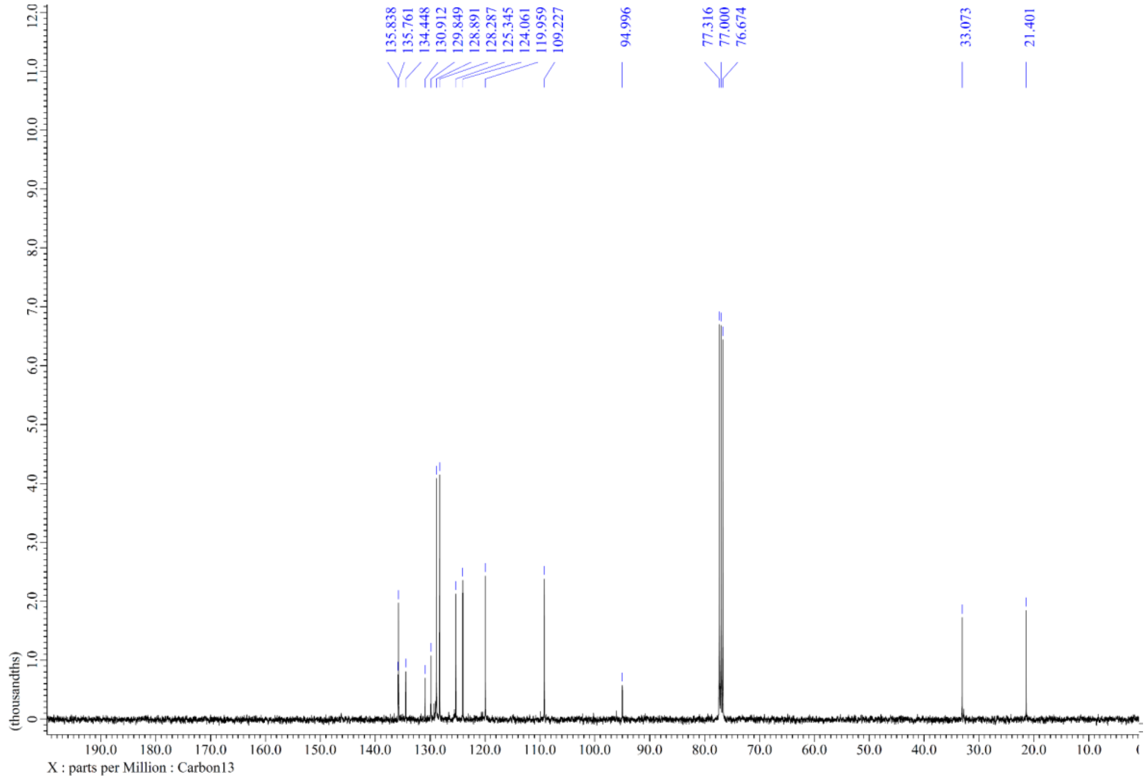

^{1}H NMR of **3da**

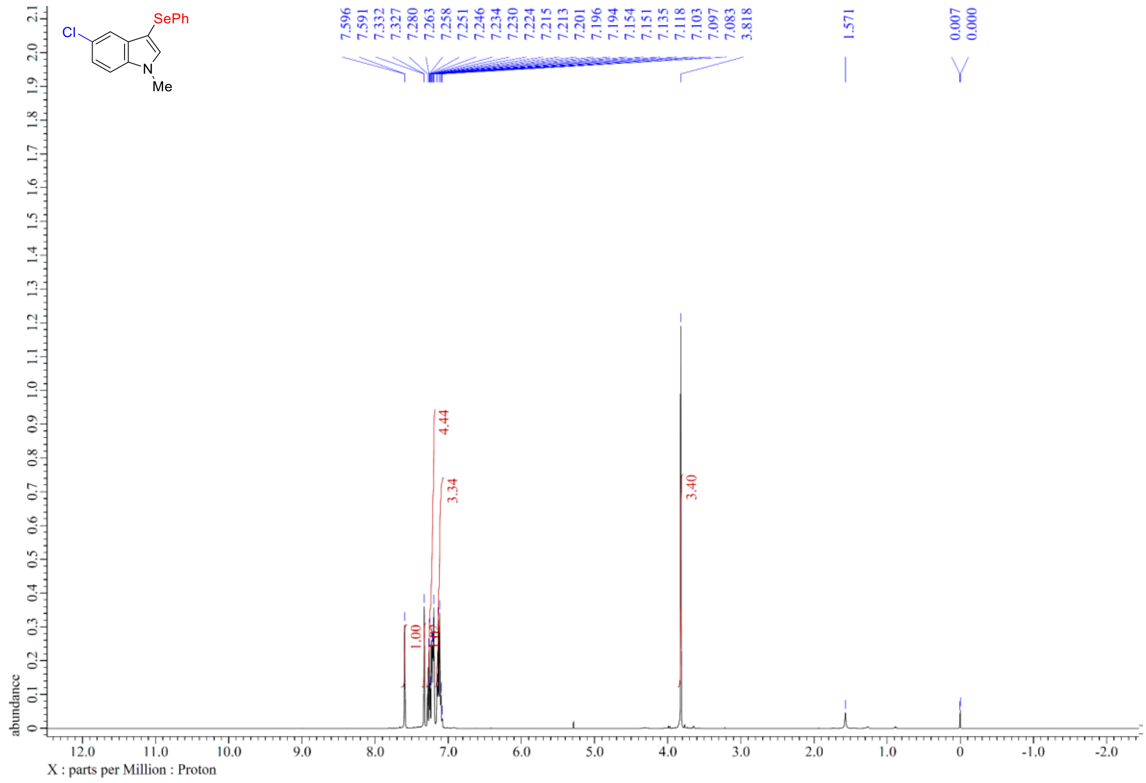

^{13}C NMR of **3da**

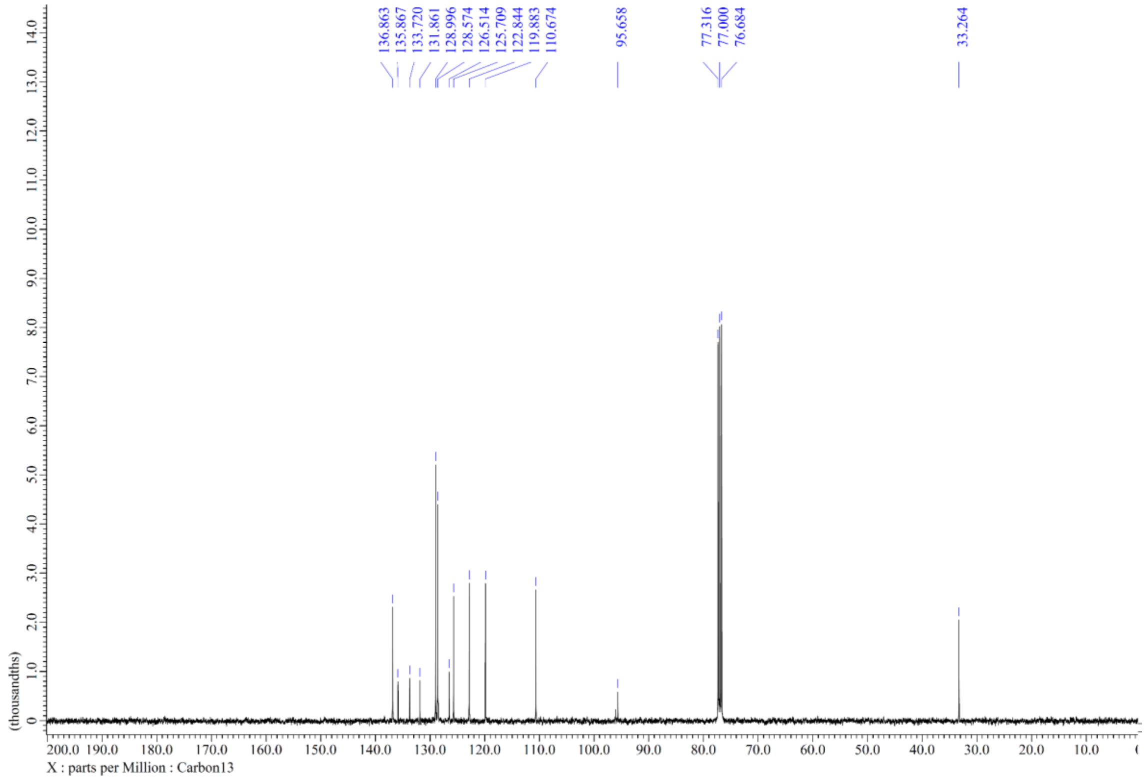

^{1}H NMR of **3ea**

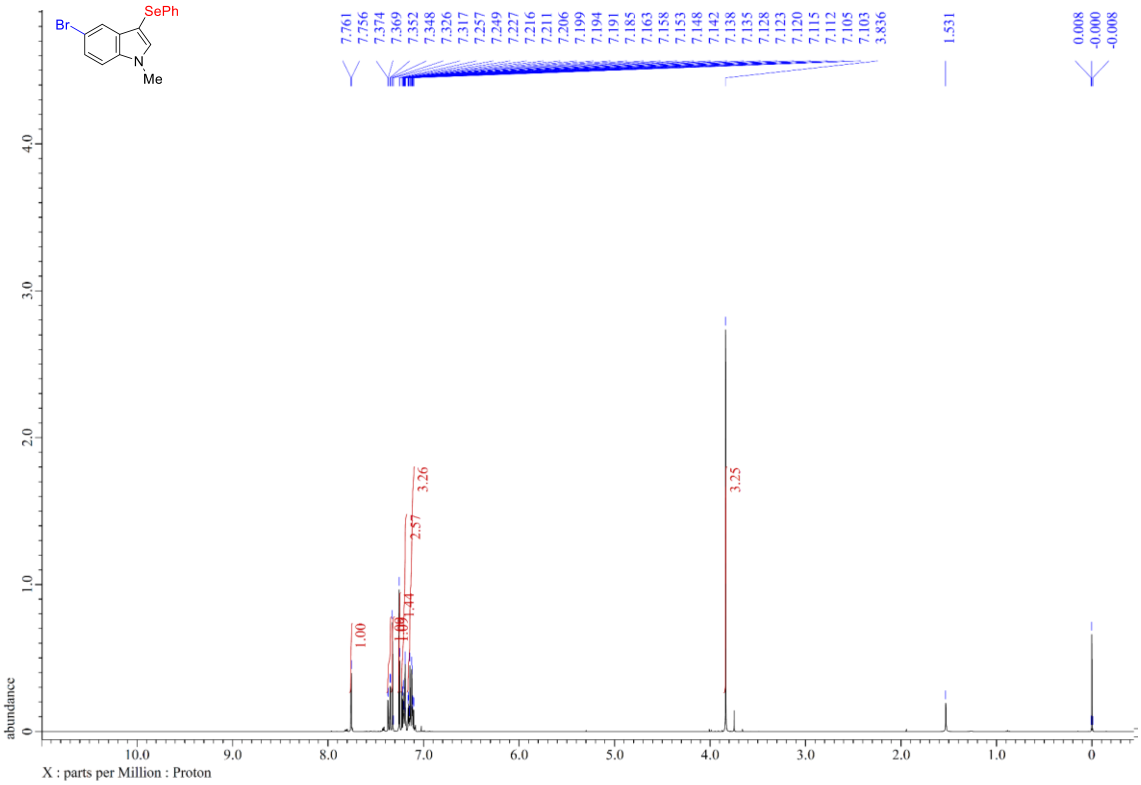

^{13}C NMR of **3ea**

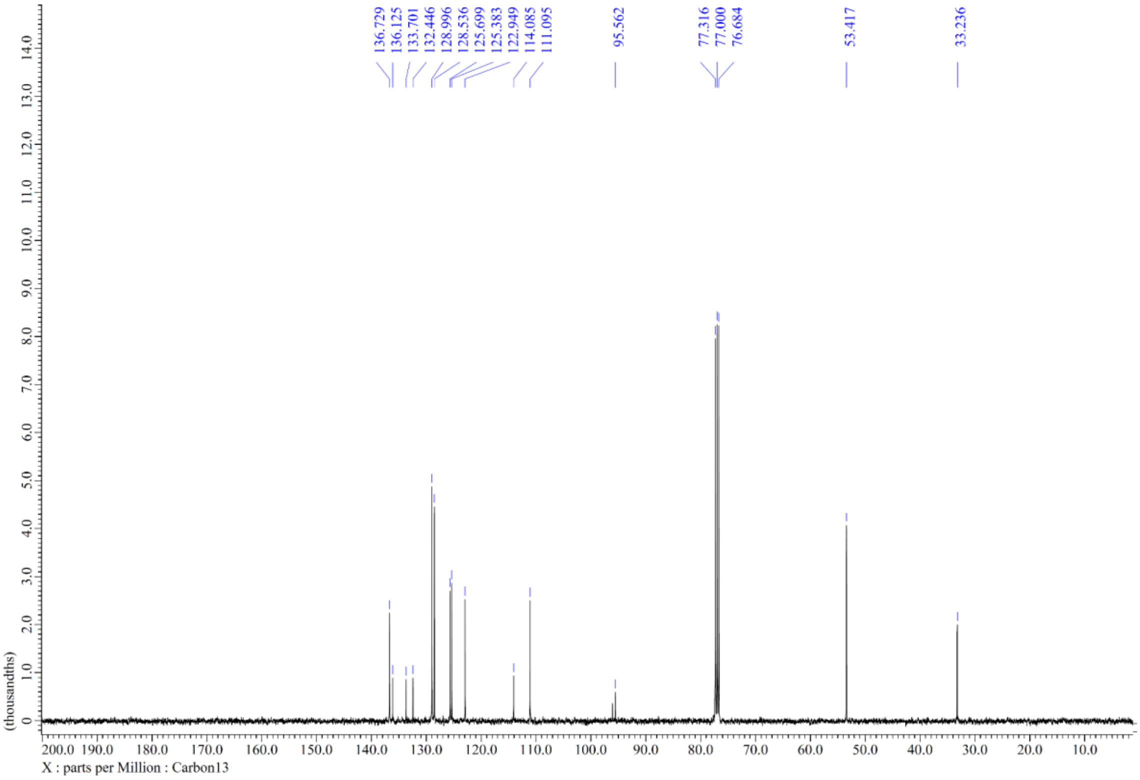

^{1}H NMR of **3fa**

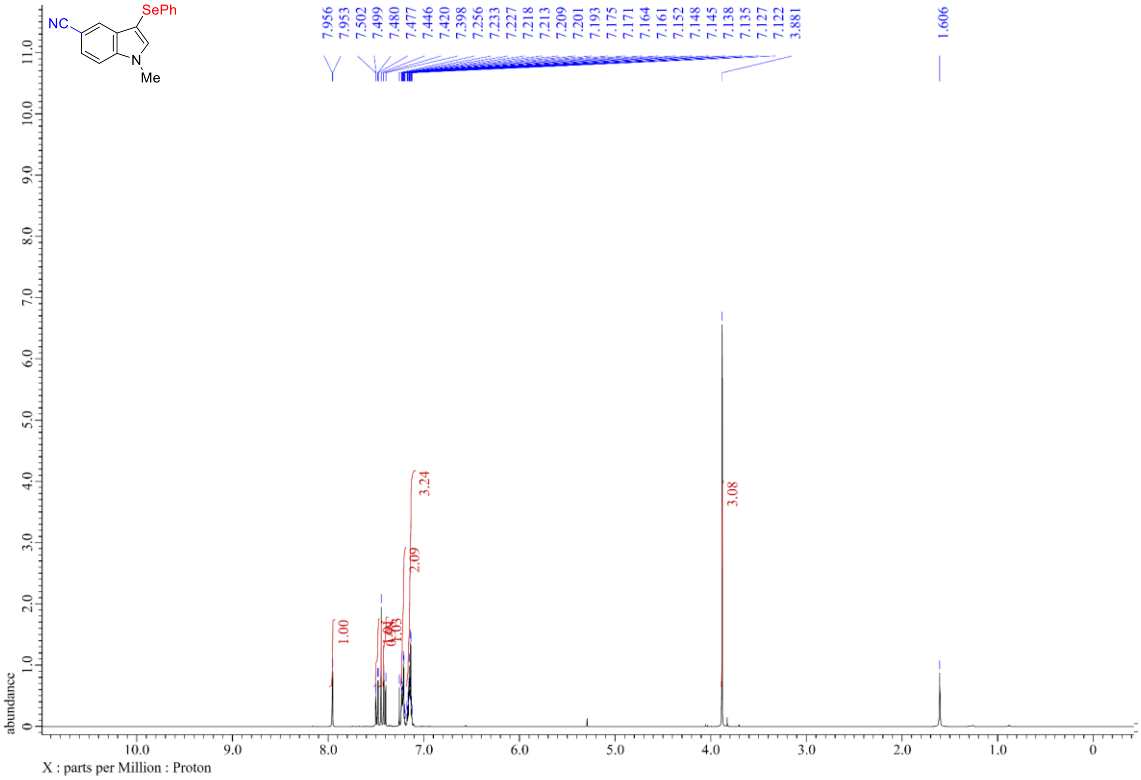

^{13}C NMR of **3fa**

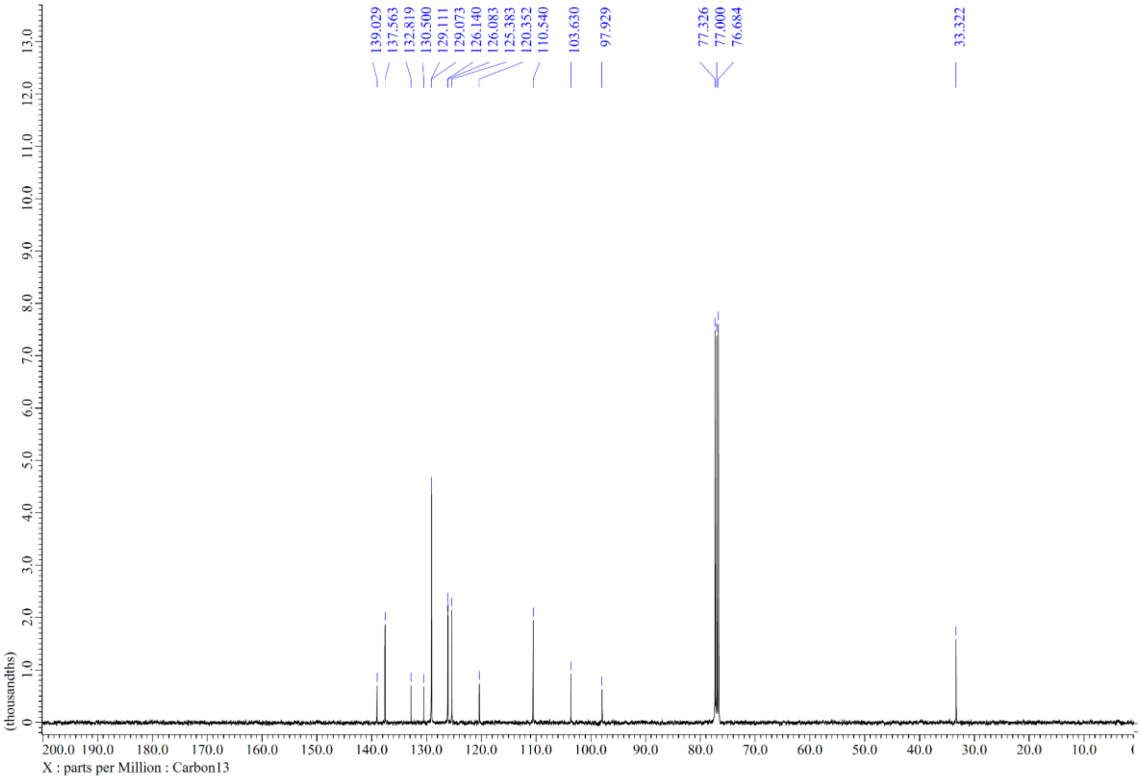

^{1}H NMR of **3ga**

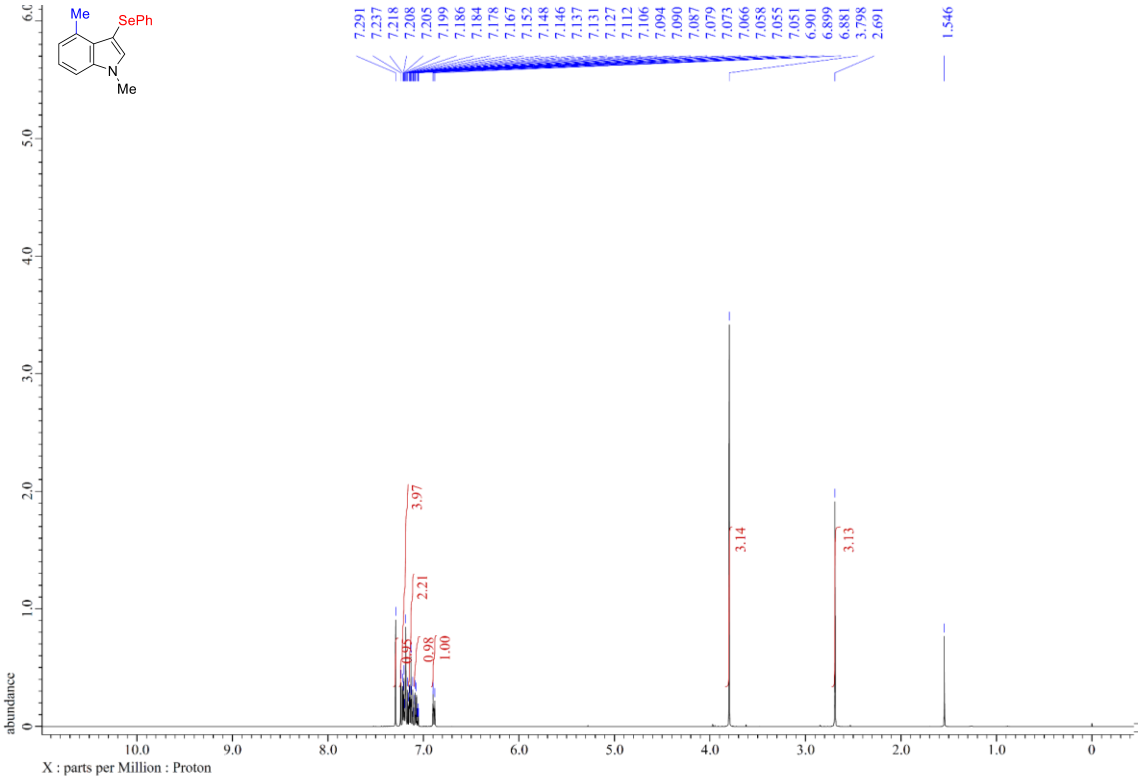

^{13}C NMR of **3ga**

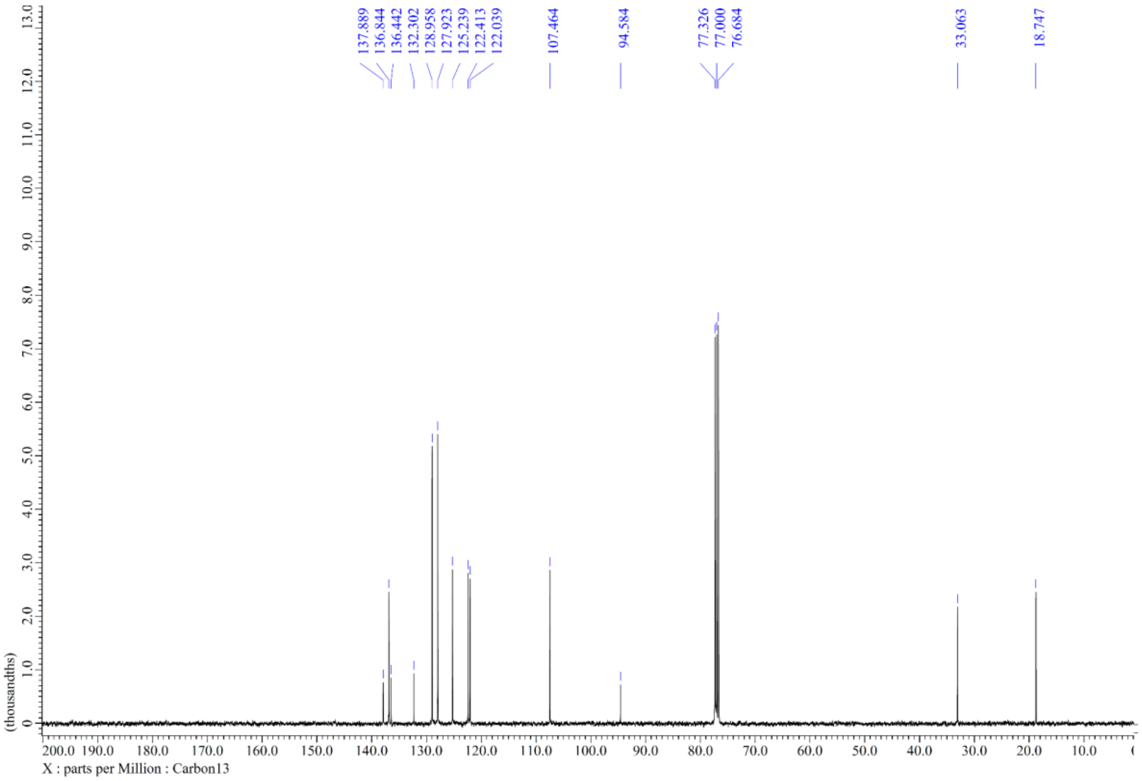

^{1}H NMR of **3ha**

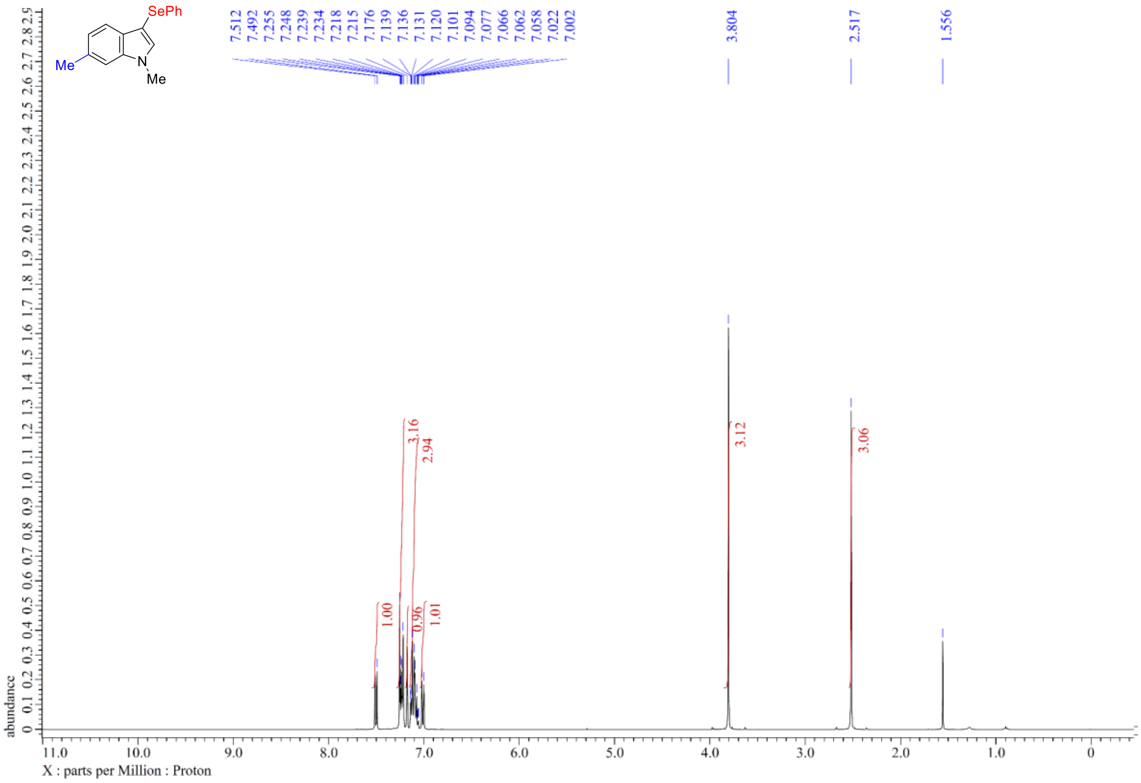

^{13}C NMR of **3ha**

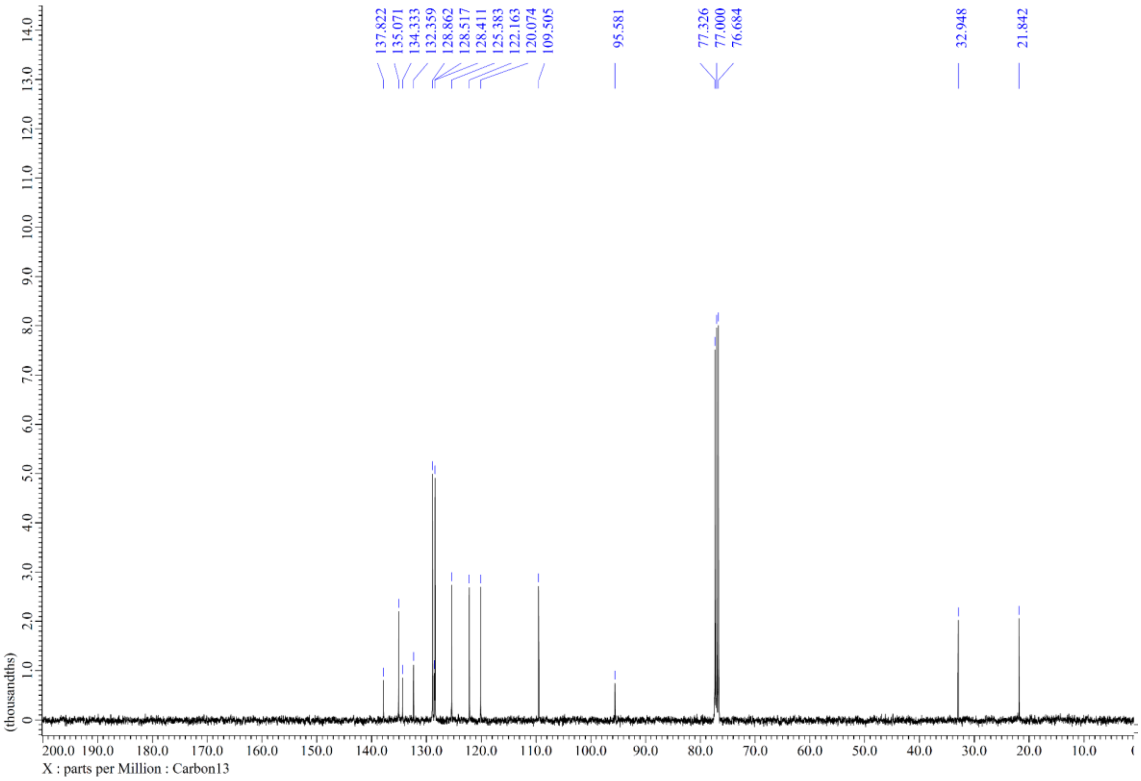

^{1}H NMR of **3ia**

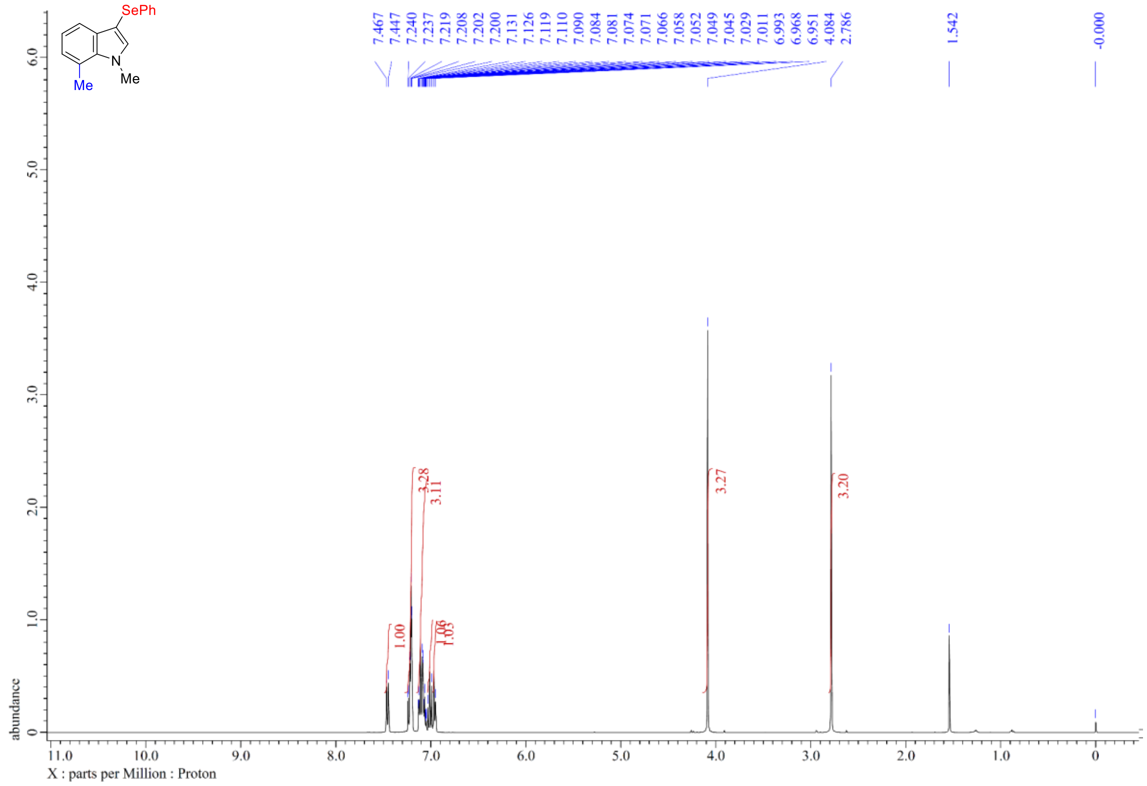

^{13}C NMR of **3ia**

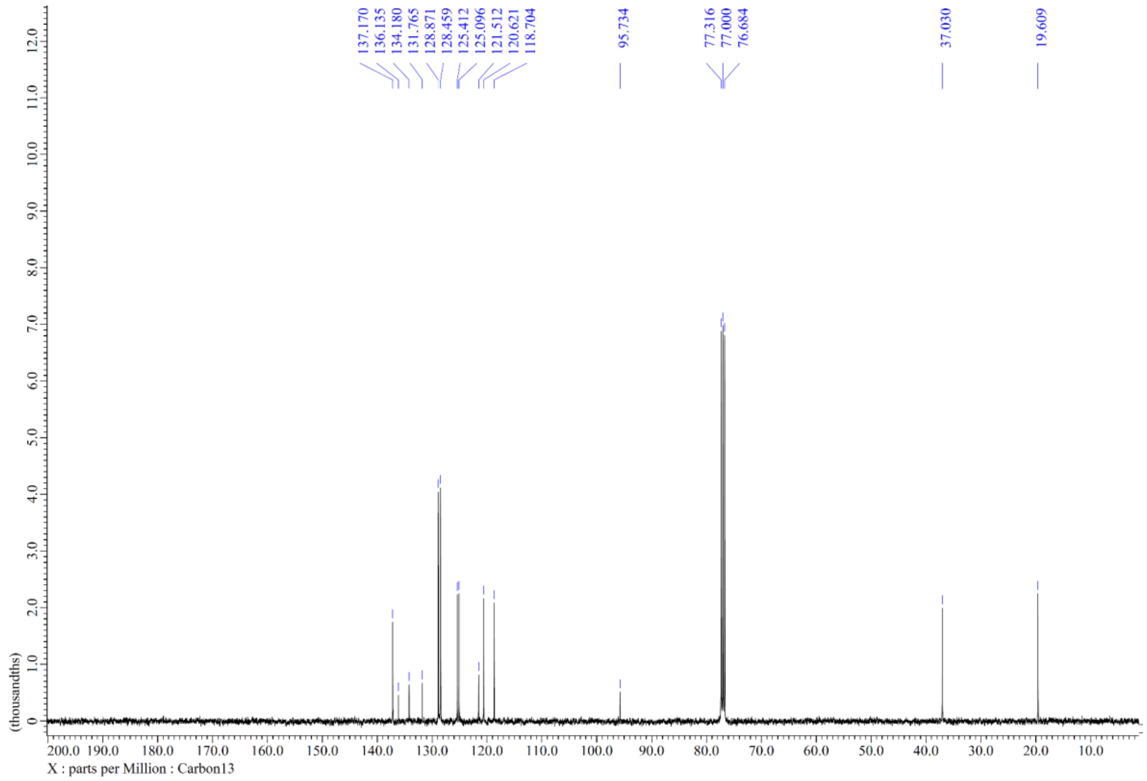

^{1}H NMR of **3ja**

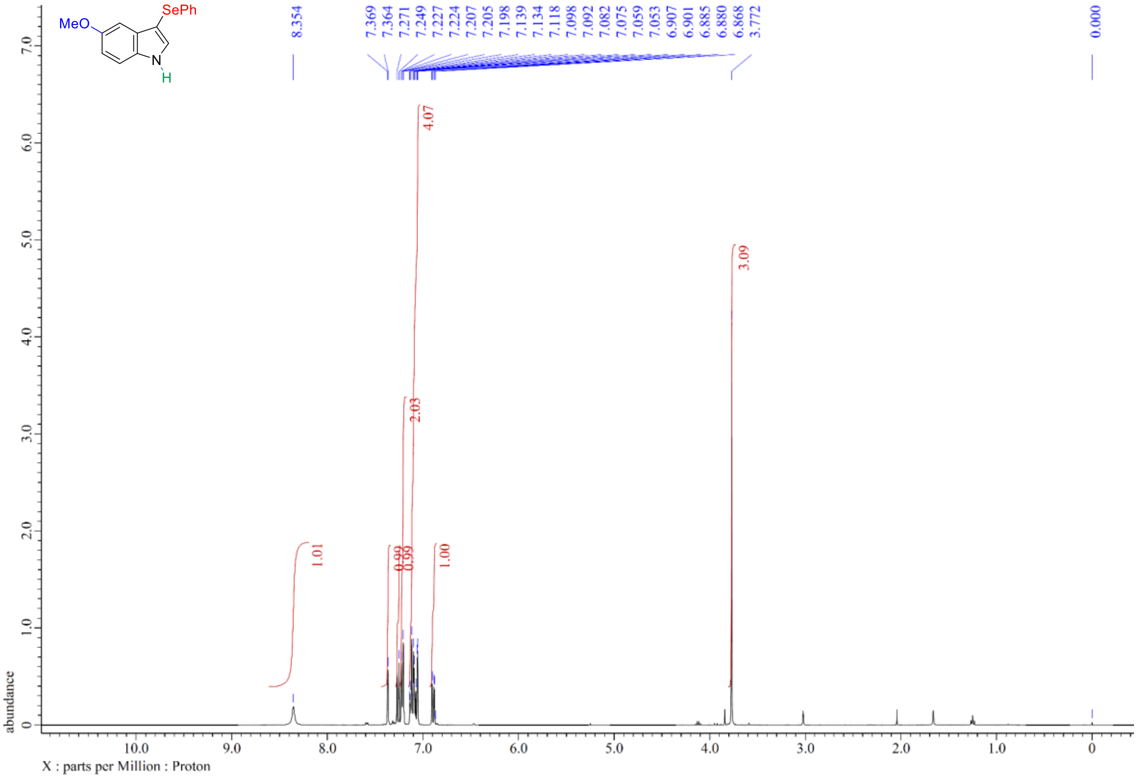

^{13}C NMR of **3ja**

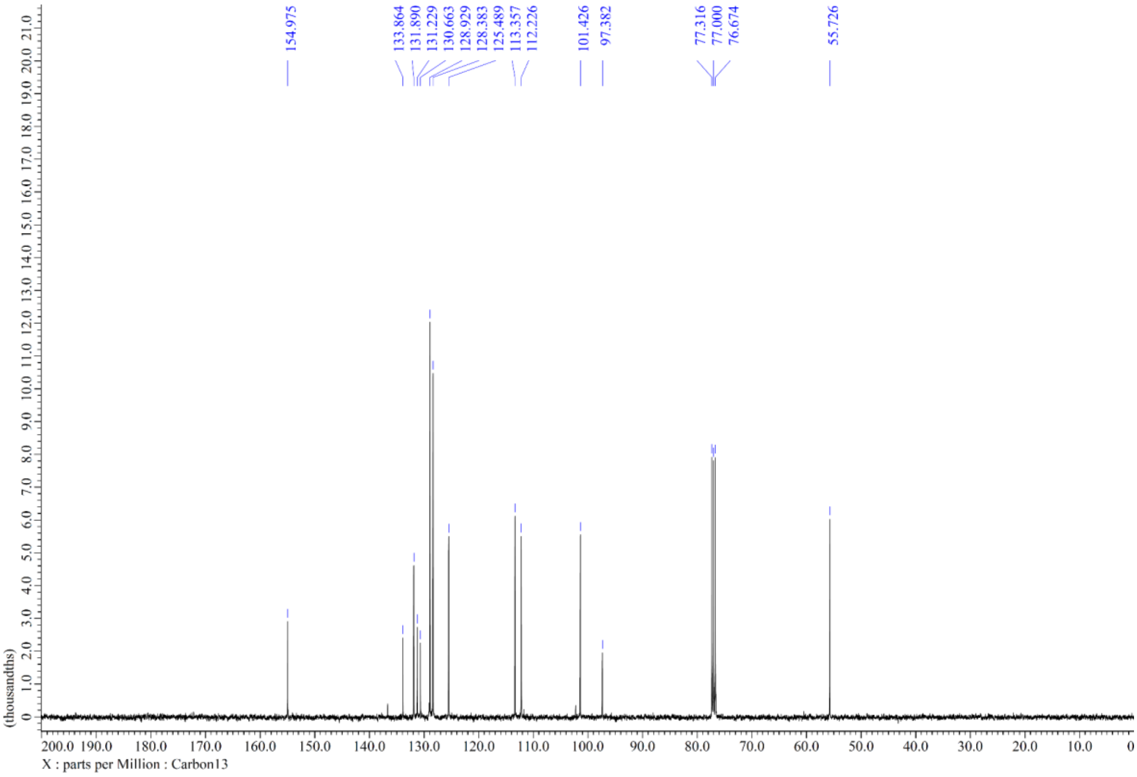

^{1}H NMR of **3ka**

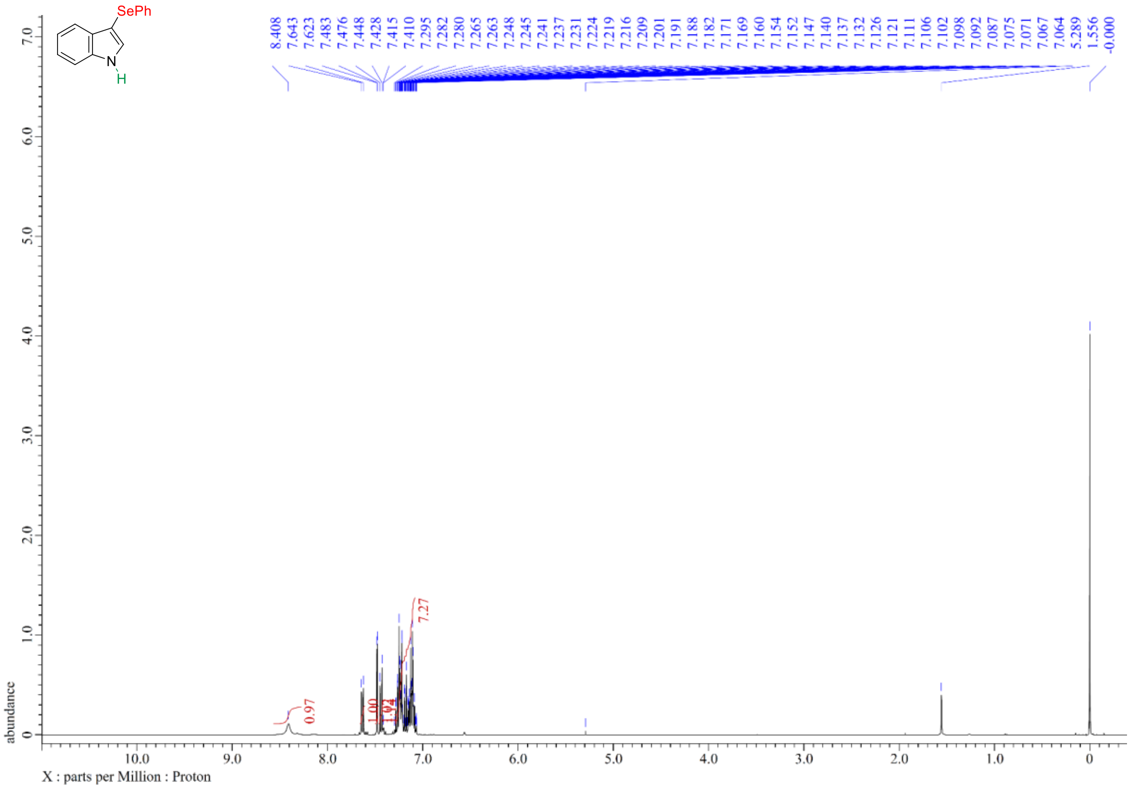

^{13}C NMR of **3ka**

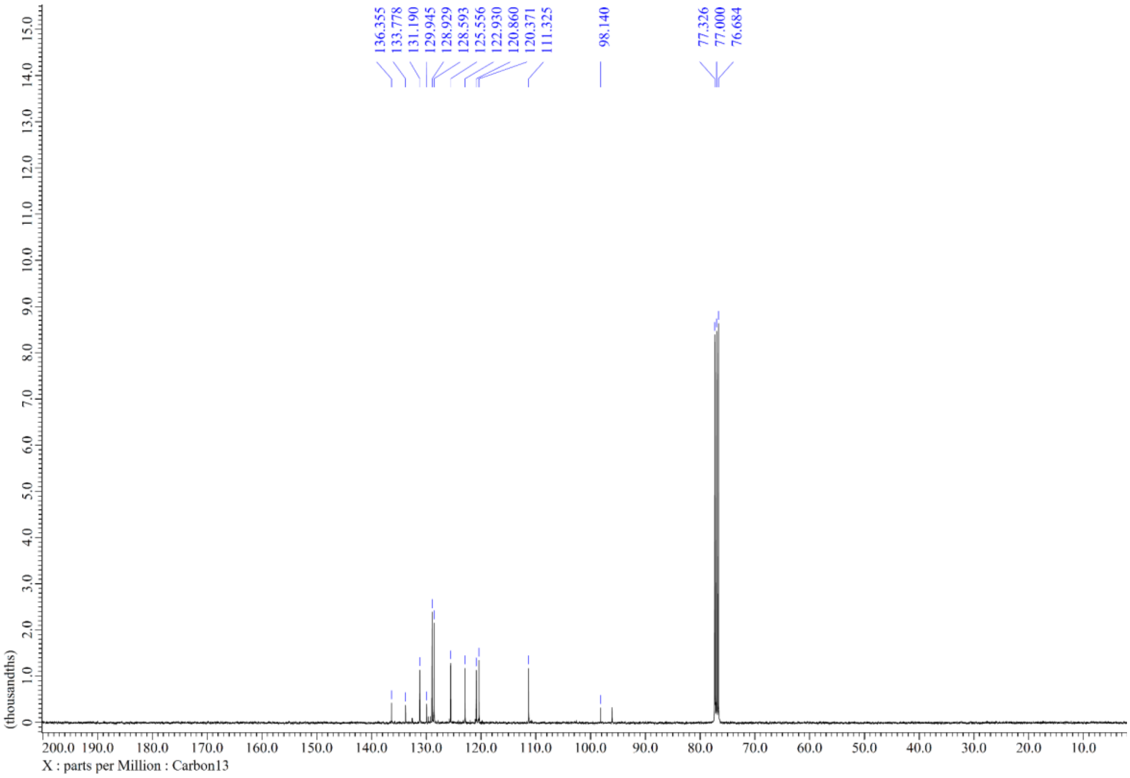

^{1}H NMR of **3la**

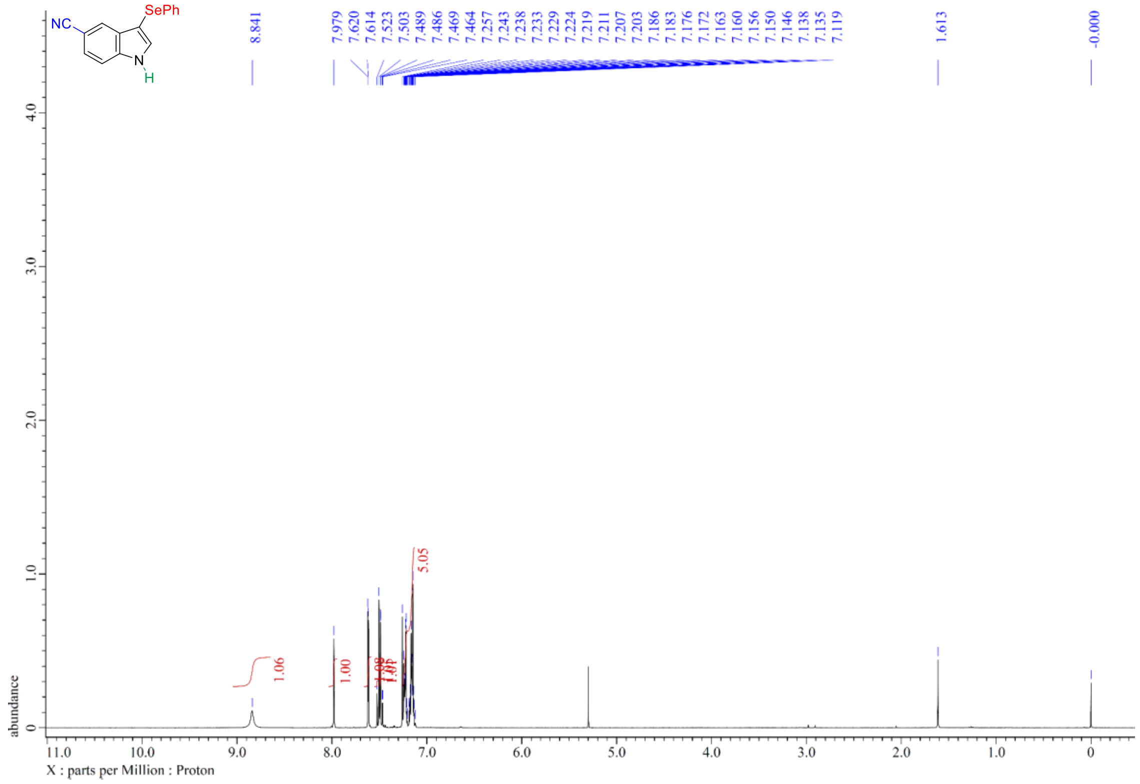

^{13}C NMR of **3la**

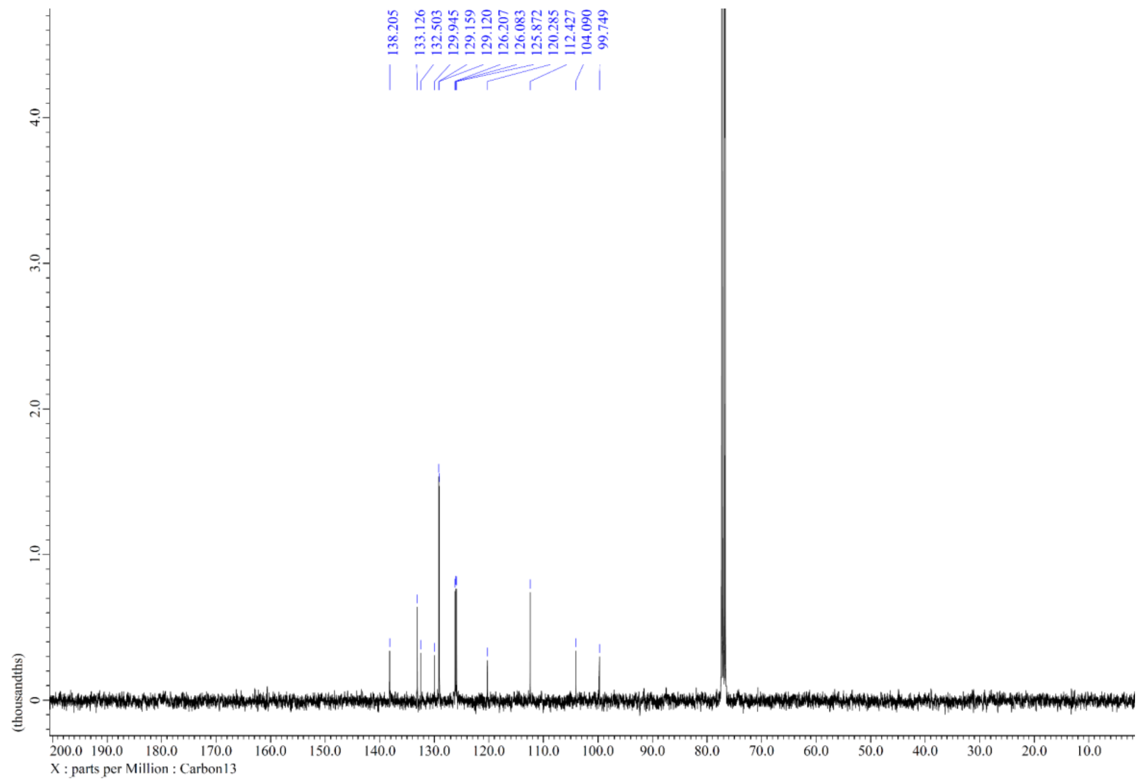

^{1}H NMR of **3ma**

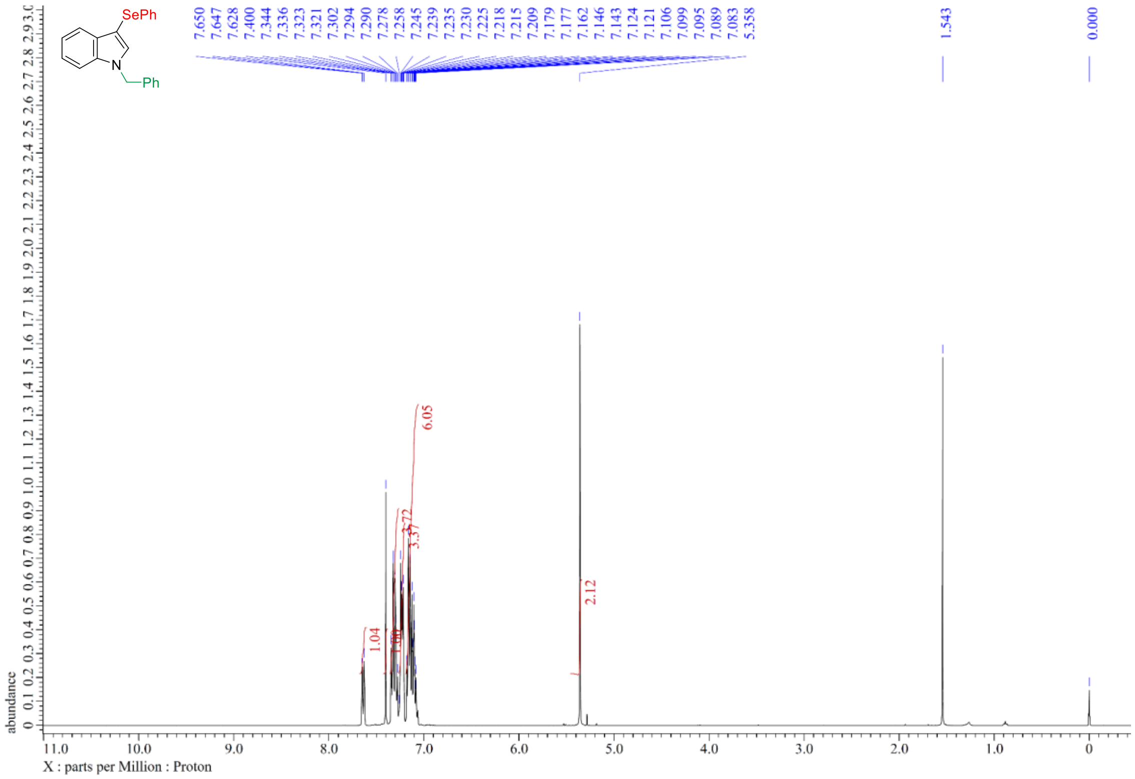

^{13}C NMR of **3ma**

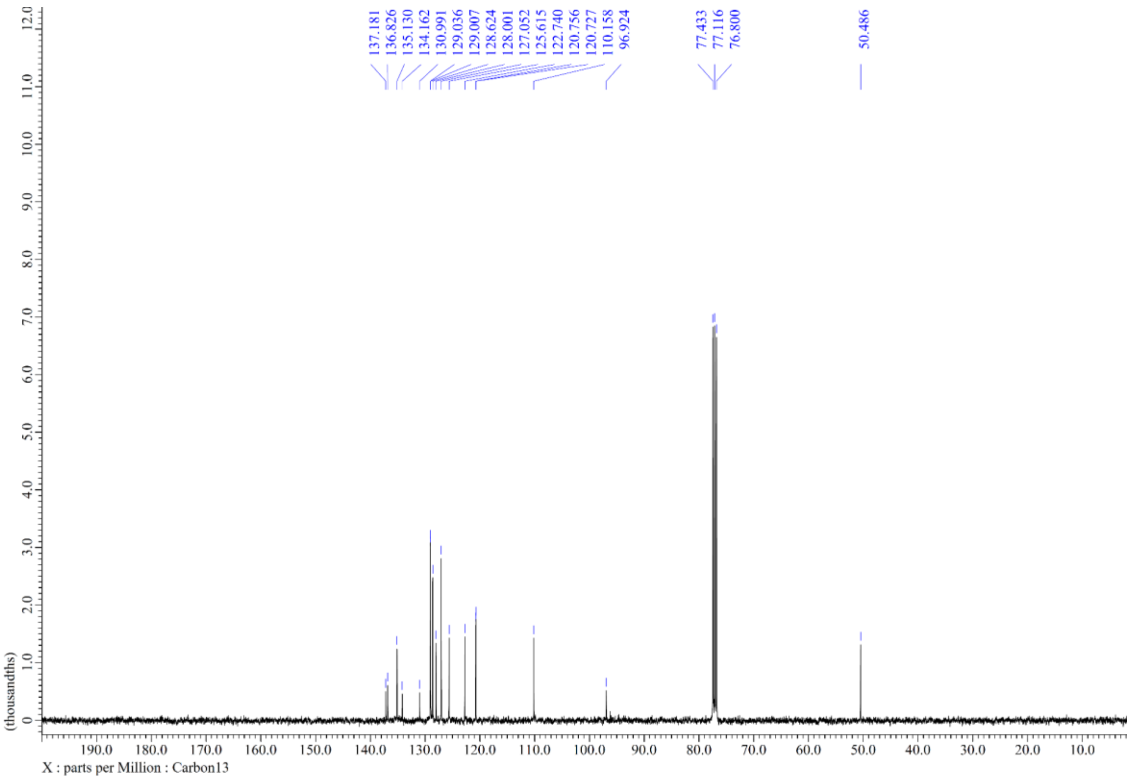

^{1}H NMR of **3na**

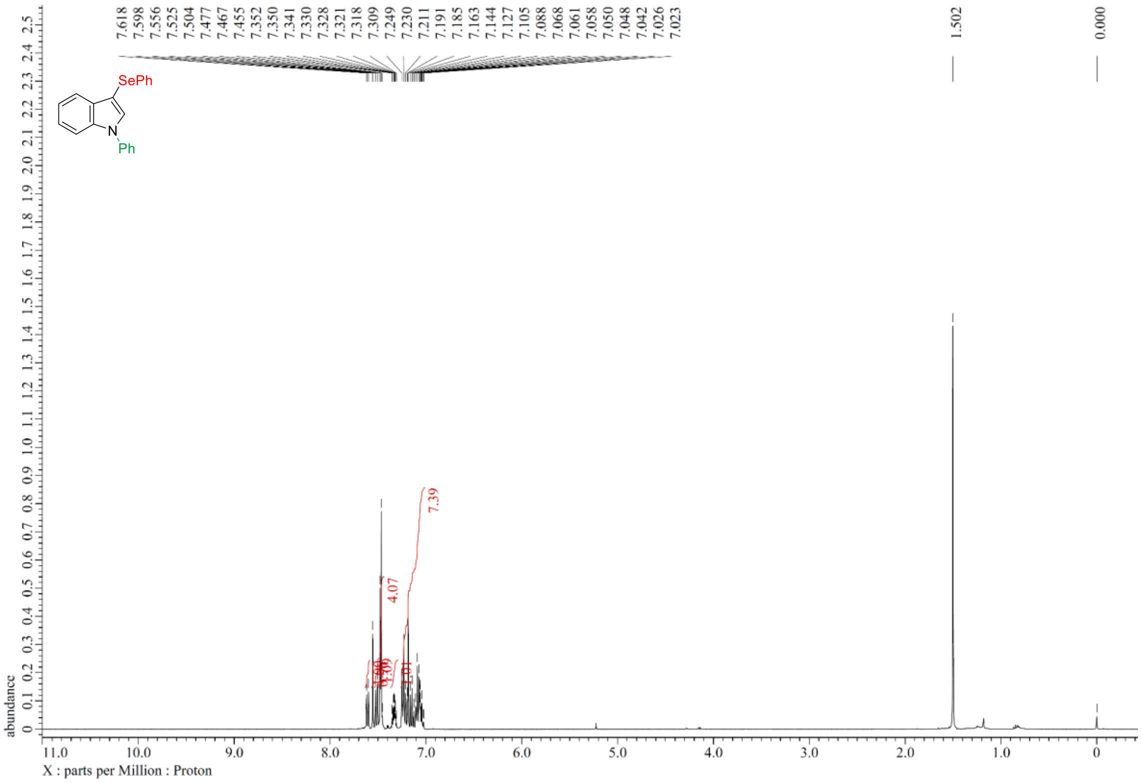

^{13}C NMR of **3na**

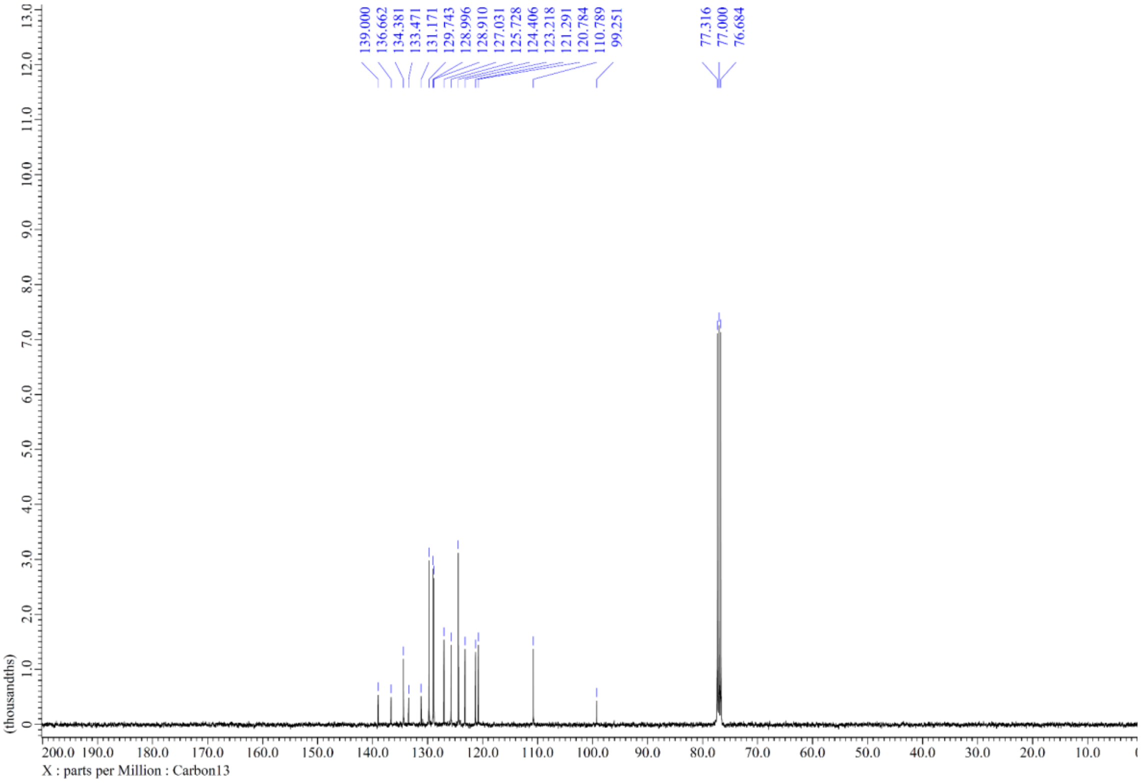

^{1}H NMR of **3pa**

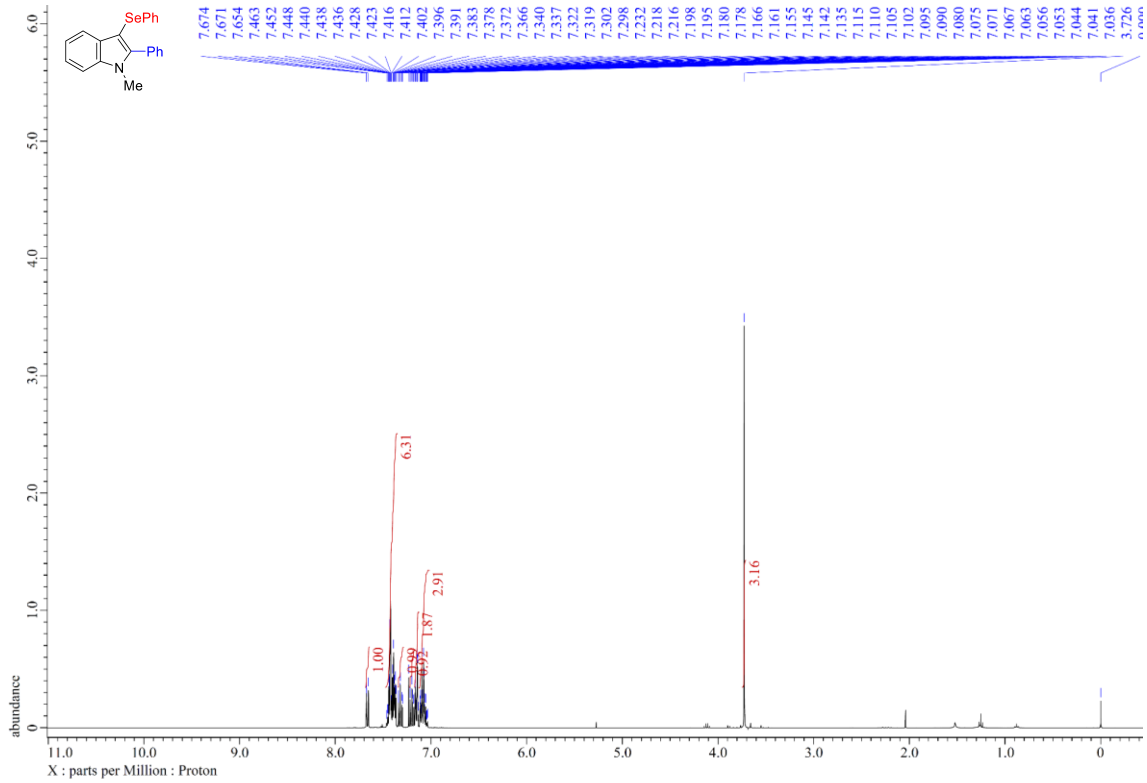

^{13}C NMR of **3pa**

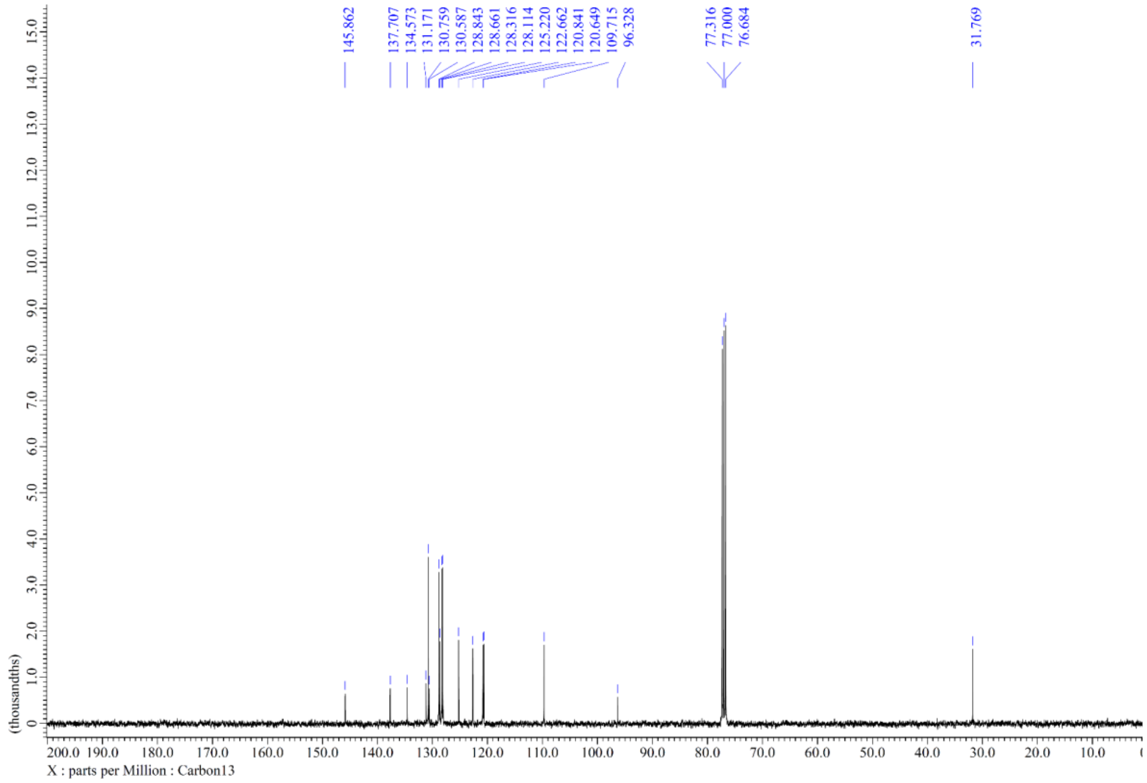

^{1}H NMR of **3qa**

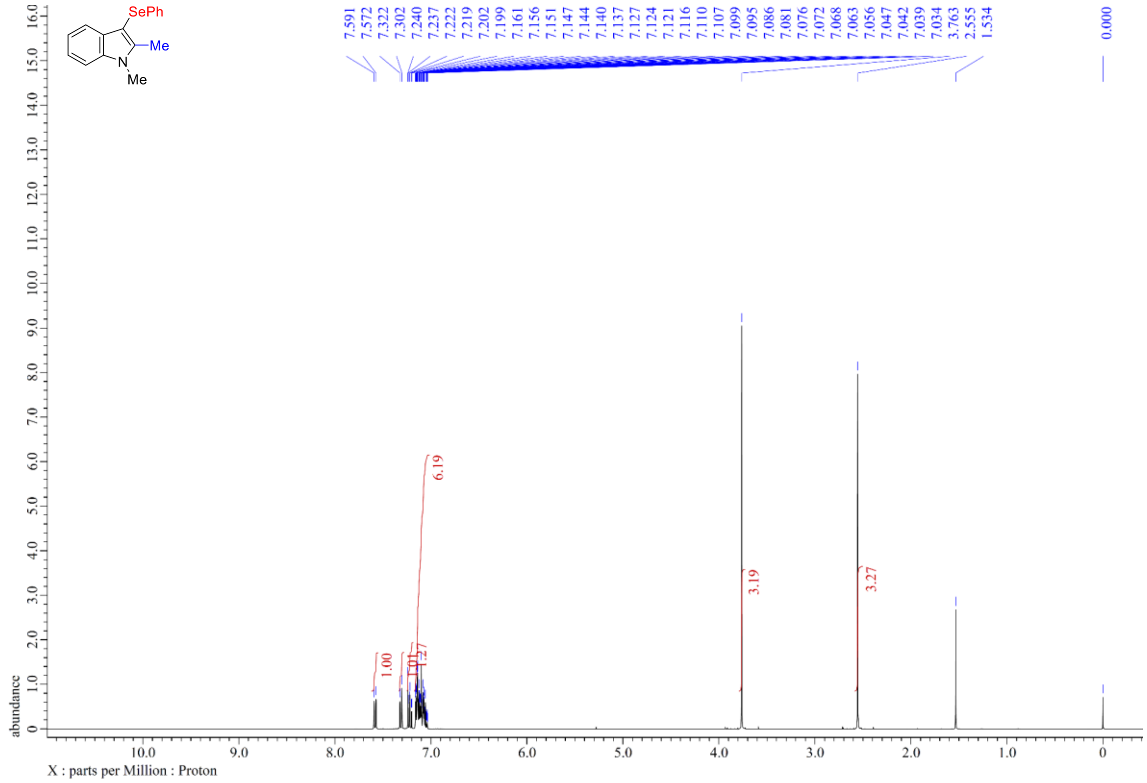

^{13}C NMR of **3qa**

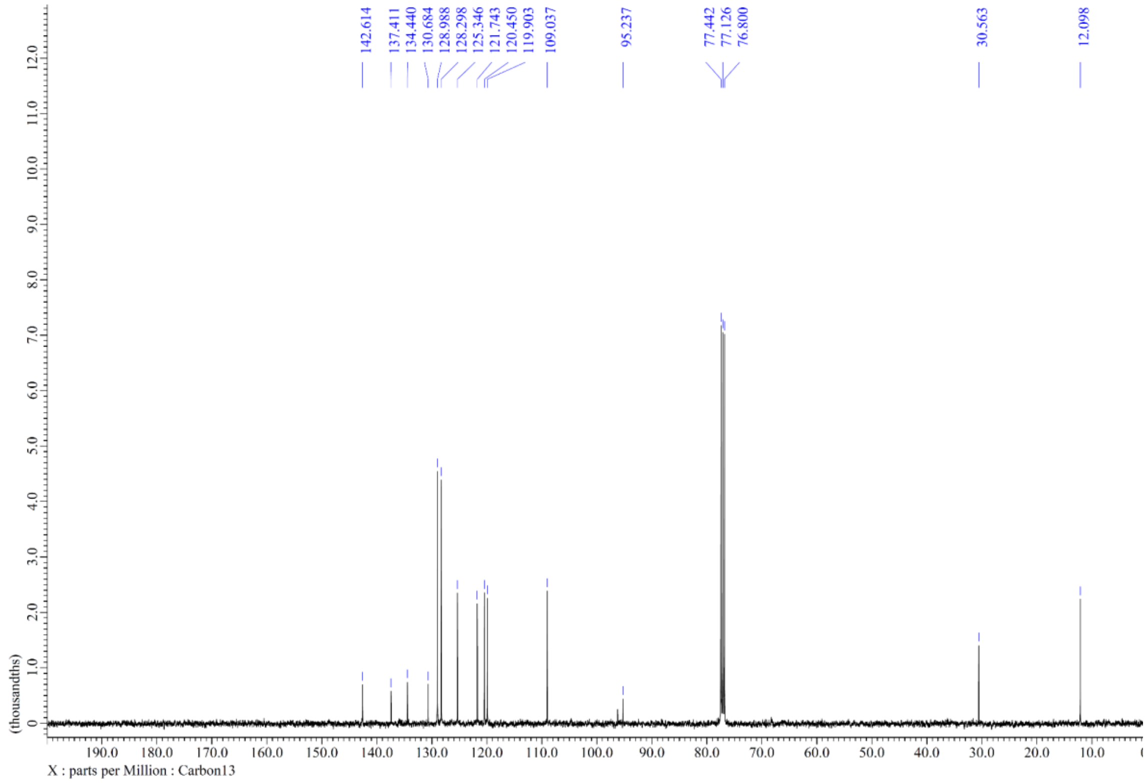

^{1}H NMR of **4**

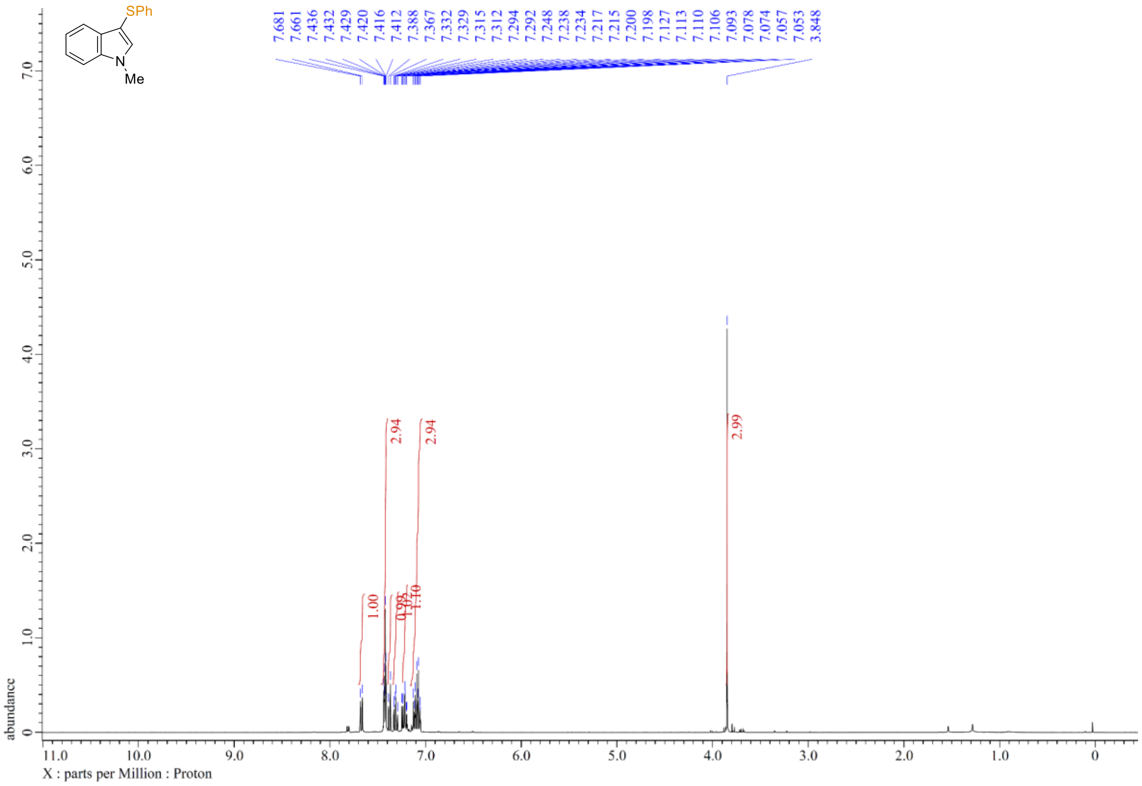

^{13}C NMR of **4**

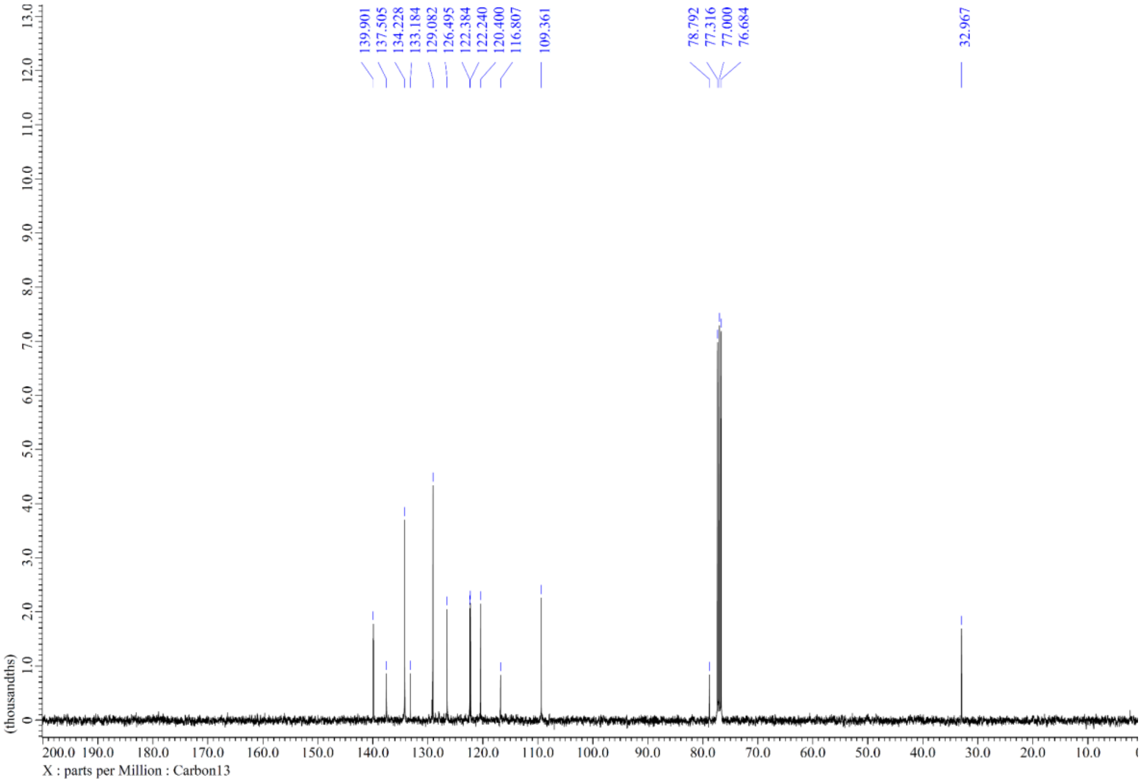

^{1}H NMR of **5**

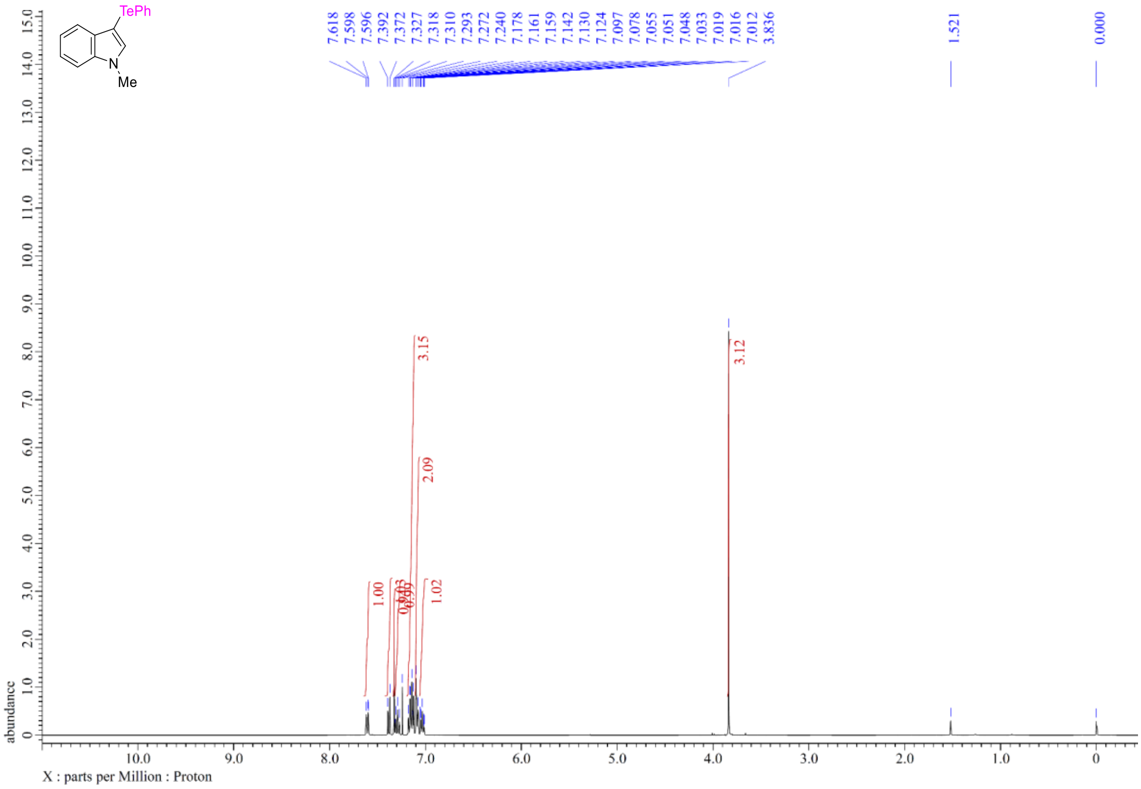

^{13}C NMR of **5**

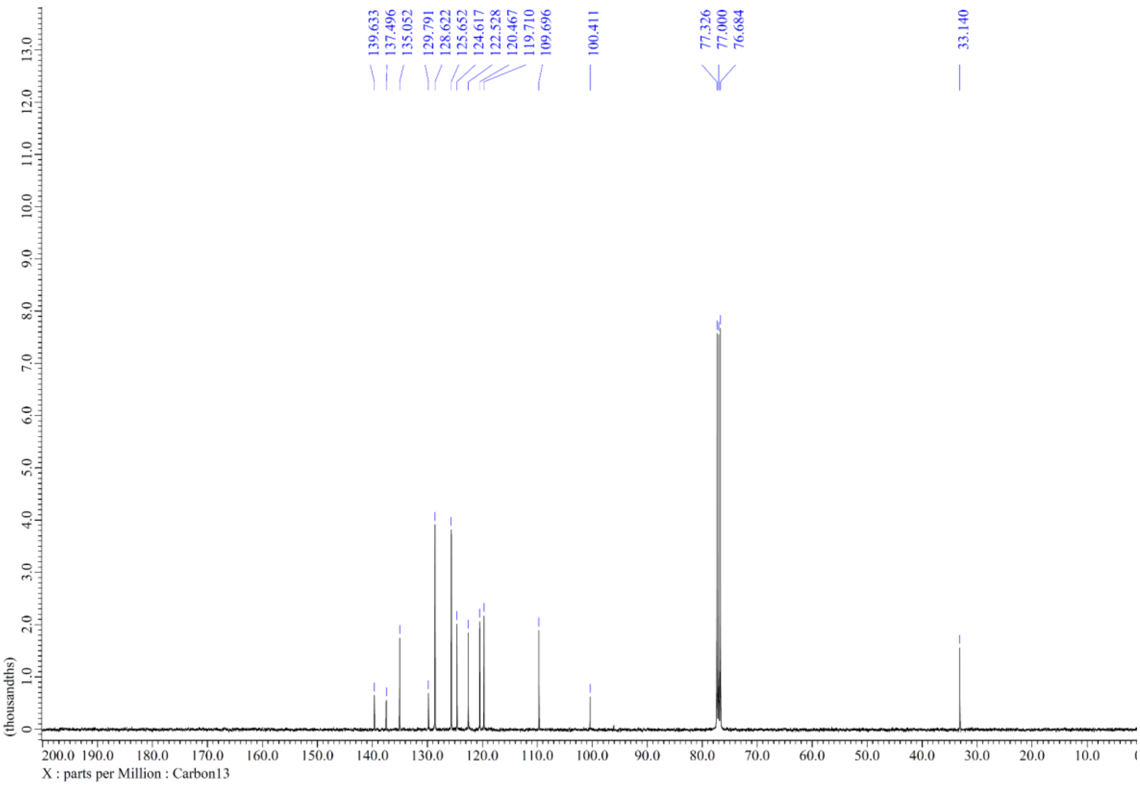

Supplement: Supplementary file 1 [file molecules-29-03227-s001.zip › SI_20240627.pdf]
